# Extracellular vesicle-associated IGF2BP3 tunes Ewing sarcoma cell migration and affects PI3K/Akt pathway in neighboring cells

**DOI:** 10.1038/s41417-023-00637-8

**Published:** 2023-06-23

**Authors:** Caterina Mancarella, Veronica Giusti, Giulia Caldoni, Maria Antonella Laginestra, Alessandro Parra, Lisa Toracchio, Giorgia Giordano, Laura Roncuzzi, Manuela Piazzi, William Blalock, Marta Columbaro, Alessandra De Feo, Katia Scotlandi

**Affiliations:** 1 Laboratory of Experimental Oncology, IRCCS Istituto Ortopedico Rizzoli, Bologna, Italy; 2 Sarcoma Unit, Candiolo Cancer Institute, FPO, IRCCS, Candiolo, Turin, Italy; 3 Department of Oncology, University of Torino, Torino, Italy; 4 Biomedical Science and Technologies and Nanobiotechnology Lab, IRCCS Istituto Ortopedico Rizzoli, Bologna, Italy; 5 Istituto di Genetica Molecolare “Luigi Luca Cavalli-Sforza”, Consiglio Nazionale delle Ricerche (IGM-CNR), Bologna, Italy; 6 IRCCS Istituto Ortopedico Rizzoli, Bologna, Italy; 7 Piattaforma di Microscopia Elettronica, IRCCS Istituto Ortopedico Rizzoli, Bologna, Italy

**Keywords:** Sarcoma, Cancer

## Abstract

Ewing sarcoma (EWS) is a challenging pediatric cancer characterized by vast intra-tumor heterogeneity. We evaluated the RNA-binding protein IGF2BP3, whose high expression correlates with a poor prognosis and an elevated tendency of metastases, as a possible soluble mediator of inter-cellular communication in EWS. Our data demonstrate that (i) IGF2BP3 is detected in cell supernatants, and it is released inside extracellular vesicles (EVs); (ii) EVs from IGF2BP3-positive or IGF2BP3-negative EWS cells reciprocally affect cell migration but not the proliferation of EWS recipient cells; (iii) EVs derived from IGF2BP3-silenced cells have a distinct miRNA cargo profile and inhibit the PI3K/Akt pathway in recipient cells; (iv) the 11 common differentially expressed miRNAs associated with IGF2BP3-positive and IGF2BP3-negative EVs correctly group IGF2BP3-positive and IGF2BP3-negative clinical tissue specimens. Overall, our data suggest that IGF2BP3 can participate in the modulation of phenotypic heterogeneity.

## Introduction

Ewing sarcoma (EWS), an aggressive mesenchymal-derived bone and soft-tissue cancer is characterized by a high tendency to form metastases. For those patients with disseminated disease at diagnosis or for those patients who do not respond to standard-of-care treatments, the prognosis is poor [1]. From a genetic point of view, EWS has one of the lowest mutational rates of all cancers [2] and is defined by a gene fusion product that is generated by one of several possible reciprocal chromosomal translocations. About 85% of cases bear the chromosomal translocation t(11;22)(q24;q12), which leads to the fusion of the gene encoding EWSR1 with that encoding Friend leukemia virus integration 1 (FLI1) [3]. Mutations of other genes, notably *STAG2* and *TP53*, have been observed in a minority of EWS at diagnosis [4, 5], supporting the idea that the fusion protein is the predominant driver of transformation in these tumors. Indeed, EWS::FLI1 depletion resulted in the inability of EWS cells to form tumors in mice [6], whereas immortalized fibroblasts forced to express EWS::ETS fusions formed tumors resembling EWS [7]. Besides acting as an aberrant transcriptional factor, EWS::FLI1 affects epigenetic control of gene expression by altering histone modifications, DNA methylation, and non-coding RNA expression. Thus, EWS::FLI1 induces an epigenetic rewiring of the genome, and it is thought to be responsible for the establishment and maintenance of subpopulations of poorly differentiated cells that display a high degree of plasticity. Several studies have revealed cell-to-cell fluctuations in EWS::FLI1 expression [8], which contribute to the marked cell-to-cell heterogeneity of EWS and its malignancy [9]. It is known that cancer cells can reversibly transition among states that differ in their competence to contribute to tumor growth or form tumors [10], thus offering a challenging but innovative perspective to deal with cancer treatments.

Besides EWS::FLI1, other molecules have been found to contribute to EWS malignancy through aberrant epigenetic control of gene expression, including CD99 and several RNA-binding proteins (RBPs) [11]. RBPs are involved in almost all aspects of post-transcriptional regulation, including editing, splicing, polyadenylation, transport, localization, RNA stability, ribosome biogenesis, and translational control. These proteins, which establish highly dynamic interactions with both coding and non-coding RNAs, as well as other proteins, are frequently deregulated in cancers and may have a major role in regulating tumor cell plasticity and phenotypic heterogeneity [12]. In particular, some RBPs including LIN28B and insulin-like growth factor 2 mRNA binding protein 3 (IGF2BP3) were found to operate in EWS::FLI1-positive cells [11, 13] and may play essential roles in EWS progression. Indeed, EWS patients with high IGF2BP3 expression in primary tumors demonstrate an unfavorable prognosis as compared to patients with low IGF2BP3 expression [14], and IGF2BP3 expression is more highly enriched in metastases as compared to localized tumors [15]. In addition, several RBPs, including hnRNPA2B1 [16], YB1 [17], HuR [18], and IGF2BP1 [19], were identified in tumor-derived extracellular vesicles (EVs), where they serve a role in the selective sorting of coding and non-coding RNAs into the EVs [20]. Thus, aside from acting inside tumor cells to regulate crucial biological processes, these proteins may also have a role in cellular communication. In this study, we investigated whether and how IGF2BP3 is released by EWS cells and if extracellular IGF2BP3 confers functional variation in recipient cells in terms of cell proliferation and/or cell migration.

## Results

### IGF2BP3 is released in EWS supernatants via EVs

**Fig. 1:**
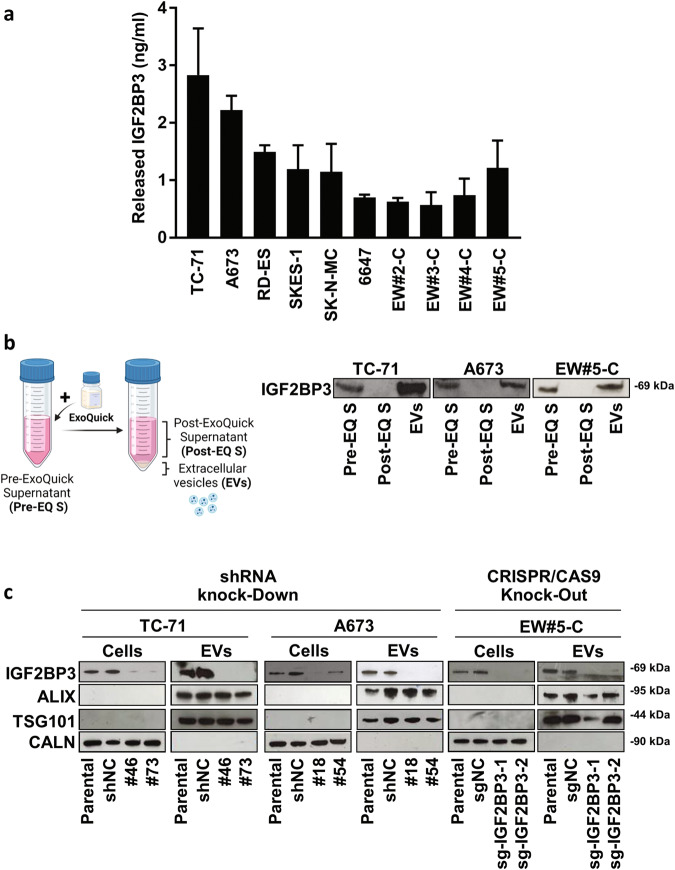
EWS cells release IGF2BP3 loaded into EVs. **a** IGF2BP3 levels in supernatants from EWS cells evaluated by ELISA assay. The bars represent the mean ± SE of two independent biological experiments with at least two replicates/each. **b** *on the left*, a schematic of the experimental plan to obtain EVs and EVs-depleted supernatant. The figure was created with BioRender.com; *on the right*, western blotting showing IGF2BP3 expression in complete supernatant (Pre-EQ S), EVs-depleted supernatant (Post-EQ S) and EVs obtained from A673, TC-71 and EW#5-C cells. Representative western blots of at least two independent experiments are shown. **c** Western blots depicting the expression of IGF2BP3 and EVs markers (TSG101 and ALIX) are shown. Protein analysis was performed on lysates from IGF2BP3-depleted (#18, #54, #46, #73, sg-IGF2BP3-1, sg-IGF2BP3-2), parental or mock-silenced (shNC and sgNC) cells and related EVs. CALN was used as a control to confirm that EVs were not contaminated with cellular material.

An ELISA assay was employed to evaluate whether IGF2BP3 is released by EWS cells. A panel of 10 human EWS cell lines was tested, including 4 cell lines established from patient-derived xenografts (PDX) that faithfully recapitulated the morphologic and genetic features of the original patient tumors [21]. IGF2BP3 was detected in the supernatants of all tested EWS cell lines at variable levels (Fig. 1a).

Since the IGF2BP3 paralogue IGF2BP1 was reported to be loaded in EVs derived from metastatic pancreatic [22] and melanoma cells [19], we examined whether IGF2BP3 was released by EWS cells into the supernatants as a free soluble molecule or loaded into EVs. IGF2BP3 was found to be present in EVs isolated from the supernatants of TC-71, A673, and EW#5-C cell lines, using the ExoQuick-TC system, but not in EV-depleted supernatants (post-ExoQuick, Post-EQ S) (Fig. 1b). As confirmation, we took advantage of IGF2BP3 knock-down models generated in TC-71 and A673 cells, using shRNA approaches [14] and a newly generated, CRISPR/CAS9-mediated IGF2BP3 knock-out model in EW#5-C cells (Supplementary Fig. 1a, b). EVs were isolated from control and IGF2BP3 knock-down or knock-out EWS cells and characterized by Nanosight nanoparticle-tracking analysis (Supplementary Fig. 2 and Supplementary Table 1), transmission electron microscopy (TEM) (Supplementary Fig. 3), and western blotting (Fig. 1c).

Nanoparticle tracking analysis revealed that the isolated EVs ranged from 100 to 180 nm in size, within the acceptable size range for EVs (Supplementary Fig. 2, Supplementary Table 1), as also indicated by western blotting of exosomal protein markers ALIX and TSG101 (Fig. 1c). Expression of IGF2BP3 was detected in the cell lysates and in the EVs from parental and mock-silenced controls but not in the EVs from IGF2BP3-deprived cells (Fig. 1c), indicating that IGF2BP3 is actively secreted and loaded into EVs according to the expression levels of the donor cells. The endoplasmic reticulum marker Calnexin (CALN) was used as a negative control; it was abundantly detected in the cell lysates but was completely absent in the EVs, thus validating that the EVs were not contaminated with cellular material. Comparing the Nanosight data obtained from control or IGF2BP3 knock-down/knock-out EWS cells, we could not appreciate any difference in size or in the concentration of EVs isolated (Supplementary Table 1).

### EVs from IGF2BP3-positive or IGF2BP3-negative cells differentially affect migratory abilities of recipient EWS cells

We previously demonstrated that the expression of IGF2BP3 was not dependent on the expression of EWS::FLI1 and did not correlate with either the proliferation rate or the sensitivity to therapeutic drugs used in EWS but rather correlated with the migratory capability of the tumor cells and the formation of metastasis [14, 15]. In keeping with this evidence EWS#5-C cells depleted of IGF2BP3 using the CRISPR-CAS9 system showed reduced cell migration when compared to control cells (Supplementary Fig. 1b, c).

**Fig. 2:**
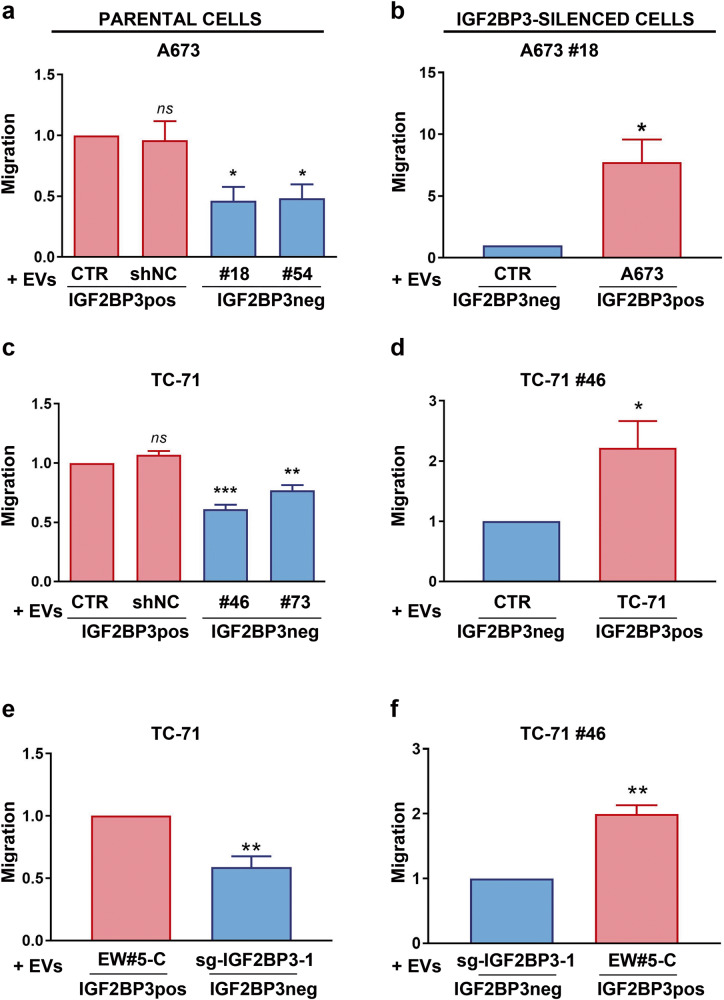
EWS cell migration after exposure to IGF2BP3pos or IGF2BP3neg EVs. EWS IGF2BP3 positive parental cells (on the left) or IGF2BP3-silenced cells (on the right) were used as recipient cells. Data from three independent experiments of cells receiving IGF2BP3pos or IGF2BP3neg EVs are shown. **a** Migration of A673 parental cells after exposure to its own EVs (CTR), or shNC EVs *versus* IGF2BP3neg EVs extracted from A673#18 or #54. Histograms represent the ratio ± SE of migrated cells compared to CTR. *p < 0.05, One-way ANOVA with respect to CTR. **b** Migration of IGF2BP3-silenced A673#18 cells after exposure to its own EVs (CTR) *versus* IGF2BP3pos EVs extracted from the parental A673 cells. Histograms represent the ratio ± SE of migrated cells compared to CTR. *p < 0.05, Student *t*-test. **c** Migration of TC-71 parental cells after exposure to its own EVs (CTR), or shNC EVs *versus* IGF2BP3neg EVs extracted from TC-71#46 or #73 cells. Histograms represent the ratio ± SE of migrated cells compared to CTR. **p < 0.01,***p < 0.001, One-way ANOVA with respect to CTR. **d** Migration of IGF2BP3-silenced cell line TC-71#46 after exposure to its own EVs (CTR) *versus* IGF2BP3pos EVs extracted from the parental TC-71 cells. Histograms represent the ratio ± SE of migrated cells compared to CTR. *p < 0.05, Student *t*-test. **e** Migration of TC-71 parental cells after exposure to IGF2BP3pos EVs extracted from EW#5-C cells *versus* IGF2BP3neg EVs extracted from EW#5-C sg-IGF2BP3-1 cells. Histograms represent the ratio ± SE of migrated cells compared to TC-71 treated with EW#5-C EVs. **p < 0.01, Student *t*-test. **f** Migration of IGF2BP3-silenced cell line TC-71#46 after exposure to IGF2BP3neg EVs extracted from EW#5-C sg-IGF2BP3-1 *versus* IGF2BP3pos EVs extracted from parental EW#5-C. Histograms represent the ratio ± SE of migrated cells compared to TC-71#46 cells treated with sg-IGF2BP3-1. **p < 0.01, Student *t*-test.

To understand the function of IGF2BP3 in the EVs on EWS cells, we first labeled EVs from the IGF2BP3pos TC-71 cells (CTR EVs) or from the IGF2BP3neg TC-71#46 cells (#46 EVs) with PKH67 fluorescent dye and verified their uptake into recipient cells. Endocytic structures (green) accumulated within TC-71 cells, indicating that the recipient cells had no preference in taking up EVs from either control or knock-down cells (Supplementary Fig. 4a). Interestingly, the uptake of IGF2BP3pos EVs resulted in an increase of IGF2BP3 in recipient cells (Supplementary Fig. 4b), indicating that EVs are capable of transferring IGF2BP3 to surrounding cells. As confirmation, the functional role that IGF2BP3pos and IGF2BP3neg EVs had on EWS cell proliferation, migration, and invasion was evaluated. No difference was detected with respect to cell proliferation in cells receiving EVs isolated from the media of TC-71 or A673 cells (IGF2BP3pos EVs) or from IGF2BP3 knock-down cells (IGF2BP3neg EVs) (Supplementary Fig. 4c). In contrast, EVs from the IGF2BP3 knock-down cells significantly decreased migratory and invasive capabilities of parental A673 or TC-71 EWS cells as compared with cells receiving IGF2BP3pos EVs, both in transwell migration assays (Fig. 2a, c), wound healing assays (Supplementary Fig. 5a, c) and invasion through a 3D extracellular matrix (Supplementary Fig. 6a, c). To avoid possible cell line-specific artifacts, TC-71 cells were exposed to EVs from EW#5-C IGF2BP3 knock-out cells. Importantly, a significant decrease in cell migration and invasion in the recipient cells was observed (Fig. 2e; Supplementary Figs. 5e and 6e), indicating that the effect on migration is independent of the genetic background and EWS cell line used. In addition, IGF2BP3-silenced cells regained their migratory and invasive abilities when exposed to IGF2BP3pos EVs obtained from the media of parental TC-71, A673, or EW#5-C cells (Fig. 2b, d, f; Supplementary Figs. 5b, d, f and 6b, d, f). To prove that the observed phenotypic effects are EVs-mediated, we repeated wound healing assay after the administration of complete (pre-ExoQuick, Pre-EQ S) or EVs-deprived (post-ExoQuick, Post-EQ S) supernatants (Supplementary Fig. 7). Of note, the effects on migration mediated by EVs were fully recapitulated when A673, TC-71, and TC-71#46 cells were exposed to Pre-EQ S supernatants from both IGF2BP3pos and IGF2BP3neg cells; on the contrary, Post-EQ S from IGF2BP3pos and neg cells both restrained migration of recipient cells.

Altogether these data demonstrate a paracrine action of IGF2BP3pos EVs in the modulation of EWS cell migratory and invasive capabilities, thus contributing to the phenotypic heterogeneity of EWS.

### IGF2BP3 is associated with a specific miRNA signature in EVs that directly impacts the IGF1R/PI3K/Akt pathway

**Fig. 3:**
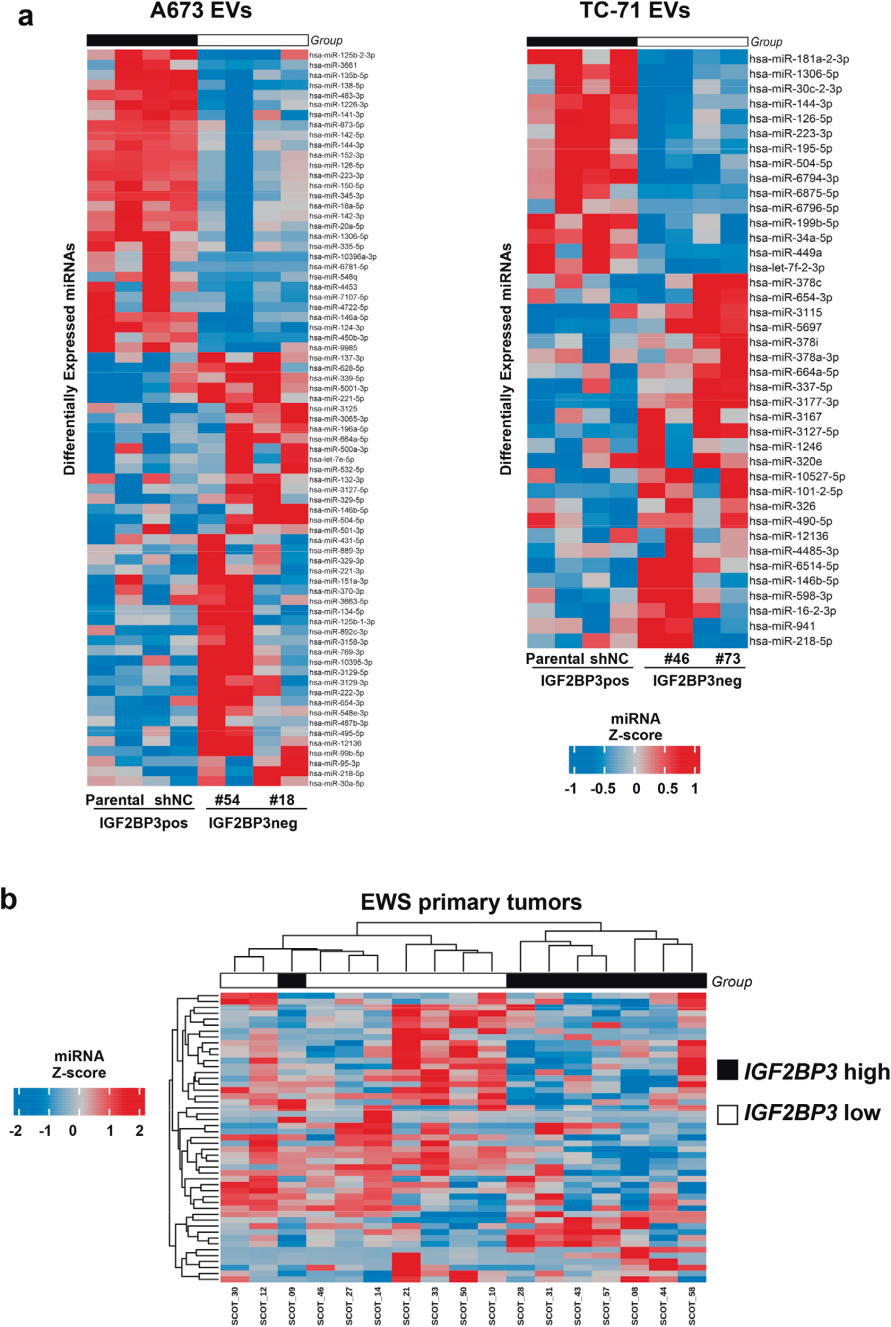
IGF2BP3 is associated with a specific miRNA signature in EVs capable to clusterize EWS patients with high or low expression of *IGF2BP3* at the tissue level. **a** Heatmap of differentially expressed miRNAs in IGF2BP3pos EVs *versus* IGF2BP3neg EVs extracted from A673 or TC-71 experimental models, respectively. **b** Heatmap unsupervised hierarchical clustering analysis of the commonly differentially expressed miRNA between IGF2BP3-EV-associated signature and those that were present in the dataset ArrayExpress accession: E-TABM-1100. The hierarchical clustering algorithm showed a distinct cluster of EWS patients with high expression of *IGF2BP3* in the tumors compared to those with low expression. In the matrix, each row represents a miRNA, and each column represents a sample. The color scale illustrates the relative expression levels (*z*-score) of miRNAs across all samples: red represents the expression level above the mean and blue represents the expression lower than the mean.

The EVs cargo of endocytic vesicles consists of proteins, lipids, microRNAs (miRNAs), and other RNA species [23]. Because many of the biological effects of EVs have been attributed to miRNAs, we decided to examine the effects of IGF2BP3 depletion on the miRNA cargo of EVs by performing miRNA expression profiling of EVs derived from parental/mock-silenced cells *versus* those derived from IGF2BP3 knock-down cells. We identified a signature of 73 differentially expressed miRNAs (DEmiR; 30 up-regulated and 43 down-regulated in parental/mock-silenced cells) in the A673 model, and a signature of 40 DEmiR (15 up-regulated and 25 down-regulated in parental/mock-silenced cells) in the TC-71 model (Fig. 3a, Supplementary Table 2).

To assess the relationship between the identified EV-derived miRNAs and IGF2BP3 expression levels in a clinical setting, we considered the A673 and TC-71 total signature of 113 DEmiR, which likely better comprises the landscape of IGF2BP3-associated miRNAs than single, cell-dependent signatures. We employed a series of 17 primary EWS cases previously profiled for miRNA expression by microarray [24] (ArrayExpress accession: E-TABM-1100) and for *IGF2BP3* expression by qRT-PCR [14]. Samples were categorized as IGF2BP3 *high* versus IGF2BP3 *low* expressors based on median *IGF2BP3* expression. Unsupervised Hierarchical Clustering allowed for the identification of EWS patients with high expression of *IGF2BP3* from patients with low expression of *IGF2BP3* (Fig. 3b). These results highlight the specific relationship of the miRNA EVs cargo with IGF2BP3 expression even in clinical samples.

To identify the functional impact of the IGF2BP3-associated miRNA signature, we considered the 11 overlapping DEmiRs between the two experimental models. Among them, miR-144-3p, miR-1306-5p, miR-223-3p, and miR-126-5p were down-regulated while miR-146b-5p, miR-218-5p, miR-654-3p, miR-3127-5p, miR-12136, and miR-664a-5p were up-regulated in the IGF2BP3neg EVs. miR-504-5p varied conversely in the two models and was not considered for further analysis. We employed miRTarBase to predict the target genes (TGs) of the 10 common DEmiRs; miR-1306-5p, miR-12136, and miR-664a-5p did not have predicted targets and were excluded from further analysis. We found 137 TGs of the remaining 7 common DEmiRs and used Cytoscape software to visualize miRNA-TGs interactions as a network (Supplementary Fig. 8).

**Fig. 4:**
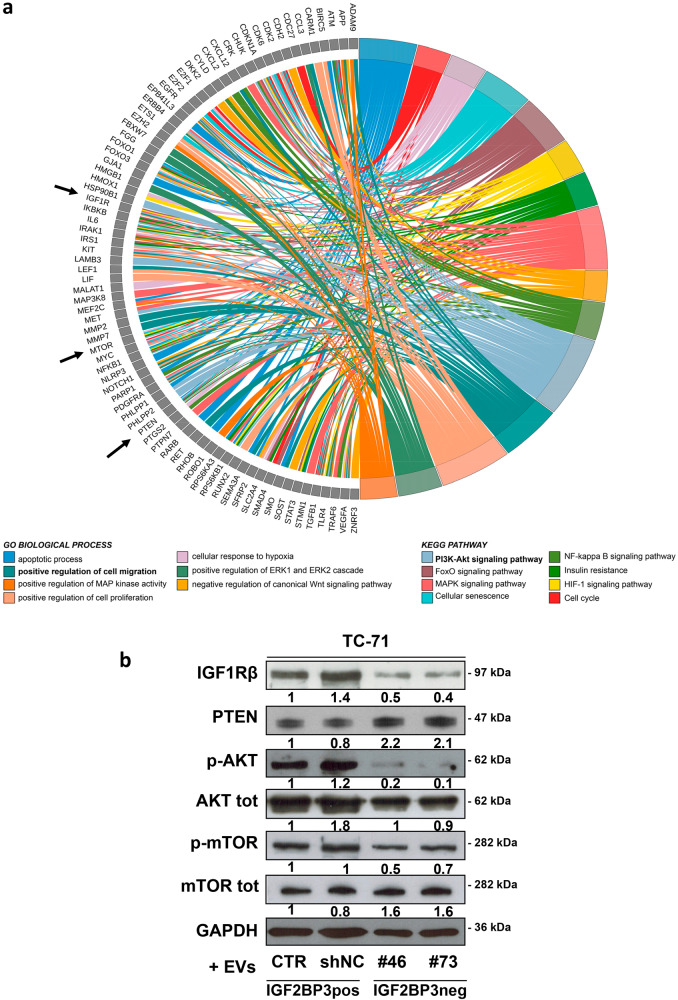
IGF2BP3 EV miRNA cargo affects IGF1R/Akt signaling. **a** Circos Plot displays the relationship between the 137 predicted target genes and the most significantly GO biological processes and KEGG pathways (Bonferroni adj-*p*value < 0.01). **b** Representative western blot depicting the expression of IGF1R, PTEN and phosphorylation of Akt and mTOR in TC-71 parental cells fused with IGF2BP3pos EVs (CTR and shNC) and IGF2BP3neg EVs (#46 and #73). GAPDH and total proteins were used as loading controls. Blots signals were quantified against GAPDH and reported as the ratio of adjusted volume optical density (OD/mm^2^) to CTR.

Functional enrichment analysis of the predicted TGs identified the regulation of cell migration and PI3K/Akt signaling among the most significant GO biological processes and KEGG pathways, respectively (Fig. 4a, Supplementary Fig. 9, and Supplementary Tables 3, 4). Among the TGs participating in the regulation of cell migration, we found *IGF1R*, a major driver of EWS aggressiveness and a previously reported target of IGF2BP3 [25]. Of note, *IGF1R* was also included as part of the PI3K/Akt pathway (Fig. 4a).

As validation, TC-71 cells were exposed to EVs from the media of control cells (IGF2BP3pos EVs) or from IGF2BP3 knock-down cells (IGF2BP3neg EVs), and the protein expression of IGF1R and the activation of down-stream Akt pathway members were evaluated by western blot (Fig. 4b). In cells, IGF2BP3neg EVs induced a dramatic reduction in the expression levels of IGF1R and active Akt (p-Akt), and mTOR (p-mTOR), along with an increase in the levels of PTEN as compared to IGF2BP3pos EVs (Fig. 4b).

## Discussion

Intra-tumor heterogeneity, which indicates diversity within individual tumors, has been defined at multiple levels, including single point mutations, somatic copy number alterations, epigenetic and transcriptomic changes, influencing gene expression, and other features of the tumor microenvironment. The possibility to obtain evidence from cancer cells with diverse transcriptional landscapes and functional variations that confer the ability to influence the tumor phenotype would be of great value in both research and clinical settings. EWS is characterized by epigenetic heterogeneity, a feature not well described by the cancer stem-cell model [10]. Several studies demonstrated that the EWS::FLI1 fusion transcription factor plays a significant role in altering transcription in EWS cells [1], but data also suggest that other factors collaborate with EWS::FLI1 [26–30] leading to heterogeneous regulation of genes that support cancer plasticity and affect clinical presentation, therapeutic response, and patient outcomes. Particularly, the expression of EWS::FLI1 was found to fluctuate among cells. Cells with higher EWS::FLI1 transcriptional activity express proliferative signatures, while cells with lower transcriptional activity upregulate mesenchymal gene signatures and display enhanced metastatic potential [8, 31]. Treatments effective against EWS::FLI1 “high” cells may not effectively target EWS::FLI1 “low” cells, which could generate a residual population of more aggressive EWS::FLI1 “low” cells capable of re-establishing tumors. In this context, it is important to study the impact of other epigenetic regulators and verify to what extent the phenotypic and functional properties of EWS cells undergo reversible changes when genetic modulators are affected. In this study, we focused on the modulation of IGF2BP3, an oncofetal RBP that is associated with the formation of metastases and a worse prognosis [14, 15]. We showed for the first time that IGF2BP3 is released by EWS cells and loaded into EVs. Taking advantage of different IGF2BP3 knock-down and knock-out models, we demonstrated that EV-associated IGF2BP3 mirrors the expression of the protein inside cells and that EVs derived from EWS cells deprived of IGF2BP3 were able to inhibit the migratory capabilities of IGF2BP3-positive recipient cells. In contrast, cell migration was oppositely regulated when IGF2BP3-deprived cells received EVs from parental, IGF2BP3-positive cells, indicating that IGF2BP3-associated alterations of EVs influence the tumor phenotype and spreading capacity of EWS.

As reported for other RBPs [20], IGF2BP3 may have a role in the selection of the EVs cargo by orchestrating the loading of coding and non-coding RNAs in the EVs, most likely through the recognition of specific RNA motifs by the RNAbinding domains and the formation of cellular RBP–RNA complexes, which are then transported into EVs during their biogenesis [32, 33]. In this paper, we demonstrated that IGF2BP3 is associated with a specific miRNA signature, whose predicted target genes are enriched in the biological process of cell migration and in the regulation of the PI3K/Akt pathway. The application of this signature correctly clustered tumor samples according to their expression of IGF2BP3, confirming its value in the clinical setting. The treatment of EWS parental cells with IGF2BP3neg EVs led to the disruption of PI3K/Akt signaling downstream of IGF1R. Of note, IGF1R signaling is well recognized for its role in sustaining EWS malignancy [34, 35] and was demonstrated to be required for EWS::FLI1-mediated transformation of EWS cells [36]. The EWS-associated fusion oncoproteins were found to upregulate the expression of IGF-1 and repress the expression of negative regulators of IGF1R signaling [37, 38], thus leading to constitutive stimulation of IGF1R-mediated pathways. Several preclinical studies supported the role of IGF1R in EWS tumorigenesis and metastasis [34, 39, 40]; however, the results of clinical trials with antibody- and tyrosine kinase inhibitor (TKI)-based IGF1R targeting have been disappointing in most EWS patients but demonstrated an objective response rate of approximately 10% with a favorable toxicity profile [41]. These controversial results are likely due to our still incomplete understanding of the regulation of IGF1R in cancer [42]. In this paper, we offer a new perspective indicating how EWS cells can dynamically regulate the activation of IGF1R/Akt signaling in neighboring cells through the release of EVs with or without IGF2BP3 and support the investigation of the expression of IGF2BP3 as a predictive marker of therapeutic response to anti-IGF1R agents.

**Fig. 5:**
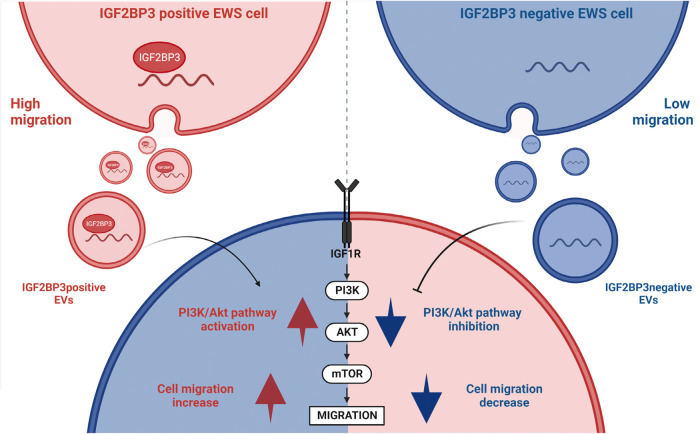
Schematic representation of IGF2BP3pos *vs* IGF2BP3neg EVs contribution to EWS phenotypic heterogeneity. IGF2BP3 positive (red) and IGF2BP3 negative (blue) EWS cells are characterized by distinct migration capabilities and release EVs with a differential cargo in terms of IGF2BP3 and miRNAs. From the functional standpoint, IGF2BP3pos vs IGF2BP3neg EVs differentially revert the phenotypic behavior of recipient cells. IGF2BP3pos EVs sustain IGF1R/Akt axis and cell migration of recipient cells while those processes are inhibited by IGF2BP3neg EVs. The figure was created with BioRender.com.

Overall, we report for the first time that IGF2BP3 is secreted by EWS cells via EVs and its presence is associated with the sorting of specific miRNAs into the vesicles. The set of miRNAs that are enriched in EVs derived from IGF2BP3neg cells was found to inhibit PI3K/Akt signaling downstream of the IGF1R in recipient cells. These IGF2BP3neg EVs inhibit EWS cell migration, while EVs from parental cells expressing IGF2BP3 were able to enhance the migration of recipient IGF2BP3neg cells (Fig. 5). This indicates that on one hand cells with high expression of IGF2BP3 can increase the malignancy of neighboring cells, while on the other, the reduction of IGF2BP3 expression has therapeutic potential with possible bystander effects. Besides acting as an oncogene inside cells, we demonstrate that IGF2BP3 can mediate reversible communications between cells, thus contributing to increased intratumor heterogeneity and cell plasticity and its detection may offer an opportunity to predict the efficacy of agents targeting the IGF system.

## Materials and methods

### Experimental models

In vitro, studies were conducted employing 6 EWS cell lines and 4 Patient-Derived Xenograft (PDX)-derived cell lines. EWS cell lines 6647 (RRID:CVCL_H722) and TC-71 (RRID:CVCL_2213) were kindly provided by T.J. Triche (Children’s Hospital, Los Angeles, CA, USA); SK-N-MC (RRID:CVCL_0530), SK-ES-1 (RRID:CVCL_0627), and RD-ES (RRID:CVCL_2169) cell lines were purchased from the American Type Culture Collection, ATCC (Rockville, MD, USA); the A673 cell line (RRID:CVCL_0080) was provided by Dr. H. Kovar (St. Anna Kinderkrebsforschung, Vienna Austria). The PDX-derived cell lines IOR_PDX-EW#2-C, IOR_PDX-EW#3-C, IOR_PDX-EW#4-C, and IOR_PDX-EW#5-C were obtained from the respective EWS PDXs [21] and characterized as previously reported [43, 44]. The PDX-derived cell lines are referred to in this study as EW#2-C, EW#3-C, EW#4-C, EW#5-C.

TC-71 and A673 cells with stable knock-down of IGF2BP3 expression using short hairpin (shIGF2BP3) were previously obtained at our laboratory [25] and maintained in a regular culture medium supplemented with puromycin 2 µg/ml (P8833, Sigma).

EW#5-C cell line was used to establish a CRISPR/CAS9 IGF2BP3 knock-out model. Chimeric single guide RNAs directed against IGF2BP3 (sgIGF2BP3) and a randomly rearranged control (sgNC), cloned into LentiCRISPR v2 plasmids (RRID:Addgene_52961) were kindly provided by Prof. A.M. Mercurio (University of Massachusetts Medical School, Worchester, MA) [45]. Co-transfection of the respective transfer and packaging plasmids, pMD2.G (RRID: Addgene_12259) and psPAX2 (RRID:Addgene_12260) into HEK293T (RRID:CVCL_0063) cells, using Lipofectamine 3000 (L3000001, Thermo Fisher Scientific) according to the manufacturer’s protocol, was performed to generate infectious lentivirus. After 48 h, the harvested culture medium was used to infect EW#5-C cells. Cells were maintained in a regular culture medium supplemented with puromycin 0.5 µg/ml (Sigma-Aldrich).

All cell lines were maintained in Iscove’s Modified Dulbecco’s Medium (IMDM; ECB2072L; Euroclone), supplemented with 10% heat-inactivated fetal bovine serum (FBS; ECS0180L; Euroclone), 20 Units/ml penicillin and 100 μg/ml streptomycin (Euroclone) in a 37 °C humidified environment at 5% CO_2_. Cells were authenticated (last control: 2017) by short tandem repeat PCR analysis (17 STRs analyzed; POWERPLEX ESX 17 Fast System, Promega) and routinary tested to exclude mycoplasma contamination (MycoAlert Mycoplasma Detection Kit, LT07-418, Lonza).

### ELISA assay

EWS cells were seeded at a density of 10,000 cells/cm^2^ in a complete medium and supernatants were harvested after 96 h. Human IGF2BP3 ELISA Kit (NBP2-82216, Novus Biological) was used to detect released IGF2BP3 expression according to manufacturer’s instructions.

### EVs purification and characterization

Cells were seeded in a complete medium at a density of 10,000–20,000 cell/cm^2^. After 24 h, cells were starved and synchronized for 5 h in IMDM supplemented with 1.5% EVs-depleted FBS. The serum was depleted of bovine EVs by Ultracentrifugation at 100,000×*g* for 6 h at 4 °C and subsequent filtration through a 0.2 µm filter prior to use. After synchronization, ultracentrifuged FBS was added up to 10%. The following day, EVs were isolated from cell culture media using ExoQuick, according to manufacturer’s instructions, or Ultracentrifugation by differential centrifugation as follows: 500×*g* for 10 min (two times), 2000×*g* for 15 min (two times), and 10,000×*g* for 30 min (two times) at 4 °C. The supernatant was then ultracentrifuged at 110,000×*g* for 1 h at 4 °C. The EVs pellet was resuspended in PBS and centrifuged at 110,000×*g* for 1 h at 4 °C (Beckman Coulter, Milan, Italy).

Collected EVs pellets were resuspended in serum-free IMDM or PBS according to down-stream analyses and quantified using the Protein Assay Dye Bradford (5000006, Bio-Rad). EVs’ concentration and size were tracked using the NanoSight NS300 system (NanoSight technology, Malvern, UK; RRID:SCR_020310), configured with a 488 nm laser and a high sensitivity sCMOS camera. NanoSight NTA software (version 3.0) was used to analyze the collected videos.

### Transmission electron microscopy (TEM)

TC-71 cells were fixed with 2.5% glutaraldehyde 0.1 M cacodylate buffer pH 7.4 for 1 h at room temperature. After post-fixation with 1% OsO_4_ in cacodylate buffer for 1 h, pellets were dehydrated in an ethanol series and embedded in Epon resin. Ultrathin sections stained with uranyl acetate and lead citrate were observed with a Jeol Jem-1011 transmission electron microscope (Jeol Inc, Peabody, MA, USA). At least 100 cells were examined for each sample.

### PKH67 staining

Labeling of EVs was performed using the fluorescent dye PKH67 according to the manufacturer’s instructions (Sigma-Aldrich, St. Louis, MO, USA). EVs internalization was evaluated in recipient EWS cells seeded on fibronectin-coated slides (3 μg/cm^2^; F1141, Sigma-Aldrich). After 3 h of exposure, cells were fixed in 4% paraformaldehyde (P6148, Sigma-Aldrich) for 10 min. Nuclei were counter-stained with Hoechst (H33258, Sigma-Aldrich) and the up-take of EVs in cells was assessed via fluorescence microscopy using a Nikon Eclipse 90i microscope (Nikon Instruments, Florence, Italy).

### Cell migration and invasion assay

Migratory abilities of cells were evaluated using Transwell chambers (Costar) with an 8 μm pore size and polycarbonate filters. 100,000 cells were seeded in IMDM + 1% FBS in the upper compartment while IMDM + 1% FBS and IGF1 (50 ng/ml, 01-208, Upstate), used as chemo attractive, were placed in the lower compartment. After 24 h of incubation migrated cells were fixed with absolute methanol, stained with Giemsa (D32884, Riedel-de-Hahn) and counted. For experiments with EVs, prior to seeding, cells were fused with isolated EVs (15 µg EVs/10,000 cells) for 30 min at 37 °C in IMDM + 1% EVs-depleted FBS. In addition, cell migration was assessed by wound healing assay. Briefly, 300,000 cells were seeded on 12-well plates coated with fibronectin (3 μg/cm^2^; Sigma-Aldrich) and allowed to grow until 100% confluence. The monolayer was scratched with a pipette tip and complete supernatants, EVs-depleted supernatants, or EVs (15 µg EVs/10,000 cells) in IMDM + 10% EVs-depleted FBS were administered. Photos were taken at time 0 and 30 h after the scratch under an inverted microscope (Zeiss, Inc., Thornwood, NY). For invasion assay, cells were fused with isolated EVs (15 µg EVs/10,000 cells) for 30 min at 37°C in IMDM + 1% EVs-depleted FBS. Then, 150,000 cells were seeded in IMDM + 1% FBS in the upper compartment of Transwell chambers (Costar) coated with 100 µl of Corning Matrigel basement membrane matrix (200 μg/ml; Corning 354234), which was incubated at 37 °C for 1 h to form a gel. IMDM + 1% FBS and IGF1 (50 ng/ml, 01-208, Upstate), used as a chemoattractant, was placed in the lower compartment. After 24 h, cells on filter’s upper surface were gently removed using cotton swabs while cells on the lower surface of the filter were fixed with absolute methanol, stained with Coomassie Brilliant Blue G-250 (#1610406, Bio-Rad), and counted under an inverted microscope (Zeiss, Inc., Thornwood, NY).

### Cell proliferation

EWS cells were seeded in 96 well plates in a complete medium. 24 h later, cells were treated with EVs (15 µg EVs/10,000 cells) in IMDM + 10% EVs-depleted FBS. Cell proliferation was evaluated after 24 h using the TACS^®^ MTT Cell Proliferation Assay kit (Trevigen, Inc., Gaithersburg, MD, USA) according to the manufacturer’s instructions.

### Western blot

Cells and EVs were lysed in radioimmunoprecipitation assay (RIPA) buffer (89900, Thermo Fisher Scientific) supplemented with protease and phosphatase inhibitors (A32959, Thermo Fisher Scientific). Cells were lysed for 30 min on ice. EVs were lysed for 10 min at room temperature. Samples were prepared in Laemmli sample buffer (1610737, Bio-Rad) and heated at 95 °C for 5 min. Samples were run on SDS gels (4568083, Bio-Rad) under denaturing conditions and blotted onto nitrocellulose membranes. The membranes were incubated overnight at 4°C with the following primary antibodies: anti-IGF2BP3 (cells 1:10,000, EVs 1:1000; MBL International Cat# RN009P, RRID:AB_1570642); anti-Alix 3A9 (1:2000; Santa Cruz Biotechnology Cat# sc-53538, RRID:AB_673821), anti-calnexin (1:2000; Cell Signaling Technology Cat# 2433, RRID:AB_2243887), anti-TSG101 (4A10) (1:2000; GeneTex Cat# GTX70255, RRID:AB_373239), anti-IGF1Rβ (F-1) (1:1000; Cell Signaling, cat# sc-390130); anti-p-Akt {Ser473} (736E11) (1:3000; Cell Signaling Technology Cat# 3787, RRID:AB_331170); anti-Akt (1:3000; Cell Signaling Technology Cat# 9272, RRID:AB_329827); anti-p-mTOR {Ser2448} (1:1000; Cell Signaling Technology Cat# 2971, RRID:AB_330970); anti-mTOR (1:1000; Cell Signaling Technology Cat# 2972, RRID:AB_330978); anti-PTEN (1:1000; Cell Signaling Technology Cat# 9552, RRID:AB_10694066); anti-GAPDH (14C10) (1:10,000; Cell Signaling Technology Cat# 2118, RRID:AB_561053). Membranes were incubated with the appropriate secondary antibody, either anti-mouse IgG-HRP (GE Healthcare Cat# NXA931, RRID: AB_772209) or anti-rabbit IgG-HRP (GE Healthcare Cat# NA934 RRID: AB_772206). The proteins were visualized with ECL Western Blotting Detection System (EMP011005, Euroclone) or SuperSignal West Pico Plus (34579/34580, Pierce). Densitometric analysis was performed by ImageJ (National Institute of Health, Bethesda, MD, USA).

### RNA-Seq sample preparation and sequencing

RNA from EVs was extracted using ExoQuick Exosome RNA Column Purification Kit (EQ808A-1, System Biosciences, CA, USA) and miRNA library synthesis was performed using the QIAseq miRNA Library Kit (QIAGEN, Germany) following manufacturer instructions (QIAseq miRNA Library Kit Handbook ver. 03/2020). Prior to sequencing, the libraries underwent electrophoretic control on DNA1000 capillary electrophoresis cartridges of Bioanalyzer 2100 (Agilent Technologies, Italia), and quantification by Qubit fluorimetric DNA High Sensitivity Assay (Thermo Fisher Scientific, USA). The libraries have been sequenced in 76 bp Single End run on a NextSeq 500 platform (Illumina, USA, RRID:SCR_017958) with automated trimming and demultiplexing made online to the sequencing with the Illumina Cloud BaseSpace Sequencing Hub.

### Processing of sequencing miRNA data and bioinformatic analyses

Raw fastq files which contain the UMI tags in the sequence (not yet extracted) were checked for read quality by FASTQC tools (http://www.bioinformatics.babraham.ac.uk/projects/fastqc; RRID:SCR_014583). Based on the structure of the entire read (the location of the 3′ adapter, UMI tag, reverse transcription primer, and 5′ adapter), we generated a regular expression to extract the UMIs tag from the sequence and placed them in the header of the reads through UMI-tool [46], (RRID:SCR_017048). Reads were aligned to Homo_sapiens.GRCh38 (Genome Reference Consortium Human Build 38, INSDC Assembly GCA_000001405.28, Dec 2013) using Burrows-Wheeler Aligner (BWA) [47] (RRID:SCR_010910). Once the alignment step was completed, we performed deduplication steps based on UMI tags to identify and remove PCR duplicates prior to any downstream analysis [46] (RRID:SCR_017048). Then, to quantify the known microRNAs in mirBase, counting the reads mapped in their loci, we employed featureCounts program [48] (RRID:SCR_012919), using Chromosomal coordinates of Homo sapiens microRNAs as an annotation file (hsa.gff3 from https://www.mirbase.org/ftp.shtml).

Differential expression analysis between EVs derived from parental/mock-silenced cells with that of EVs from IGF2BP3 knock-down cells was computed by DESeq2 R package [49] (RRID:SCR_015687). We considered differentially expressed miRNAs with a *p*-value < 0.05 and absolute |log_2_FC | =0.5 ( | FC | = 1.5). Heatmaps of differentially expressed miRNAs (*Z*-scores of log2-transformed expression values) were displayed using the ComplexHeatmap R package [50] (RRID:SCR_017270).

To test the miRNA signatures, we considered a published normalized microarray-based miRNA expression data set of 17 primary EWS cases. The patients were previously profiled for miRNA expression via microarray [24] (ArrayExpress accession: E-TABM-1100) for a list of 49 miRNA probes included in Agilent Human miRNA Microarray v.2 (Supplementary Table 5) and for *IGF2BP3* mRNA expression via qRT-PCR [14]. Unsupervised Hierachical Clustering was generated using the Hclust R function (RRID:SCR_009154) based on the Ward.D2 method and Euclidean distance as a measure of similarity (R package stats v3.6.2) and *Z*-scores of log2 transformed expression values were displayed by ComplexHeatmap R package [50] (RRID:SCR_017270).

miRNAs’ targets were searched using the miRTarBase database tool (https://mirtarbase.cuhk.edu.cn/) [51] (RRID:SCR_017355), which provides a list of experimentally validated miRNA targets and only genes predicted to be targeted by the miRNA with ‘strong evidence’ (Reporter assay or Western blot) were used for Gene Ontology (GO) and Kegg pathways analysis. GO and Kegg pathway analysis was carried out using DAVID Bioinformatics Resources (released in December 2021) according to the following constraints; count = 10 (minimum gene numbers belonging to an annotation term) and EASE value < 0.1 [52, 53] (RRID:SCR_001881). The most relevant GO/KEGG terms were selected considering Bonferroni adj-*p*value < 0.01.

To show the relationship between a list of selected miRNAs’ target genes and GO/KEGG terms we used the GOChord function implemented in the GOplot R package [54]. To visualize the miRNAs-Gene target interaction network we used Cytoscape open-source software platform [55] (RRID:SCR_003032).

### Statistics and reproducibility

All results are presented as mean ± SEM of *n* independent experiments (specified in figure legends). Differences among means were evaluated by one-way analysis of variance (ANOVA) with Tukey’s multiple comparisons test or with two-tailed Student’s *t*-test, depending on the number of groups being compared. Statistical analyses were performed using Prism version 7.0 (GraphPad Prism Software, La Jolla, CA, RRID:SCR_002798).

## Supplementary information

Supplementary Figure 1

Supplementary Figure 2

Supplementary Figure 3

Supplementary Figure 4

Supplementary Figure 5

Supplementary Figure 6

Supplementary Figure 7

Supplementary Figure 8

Supplementary Figure 9

Supplementary Table 1

Supplementary Table 2

Supplementary Table 3

Supplementary Table 4

Supplementary Table 5

## Data Availability

Microarray data are accessible through ArrayExpress accession: E-TABM-1100. miRNA-seq data are accessible through BioProject ID PRJNA942082. The original contributions presented in the study are included in the article/Supplementary Material. Further inquiries can be directed to the corresponding authors.
