# Supplementary figures and images for: Extracellular vesicle-associated IGF2BP3 tunes Ewing sarcoma cell migration and affects PI3K/Akt pathway in neighboring cells
Source: Cancer Gene Ther. 2023 Jun 23;30(9):1285–95. doi: 10.1038/s41417-023-00637-8 (PMC10501906; doi:10.1038/s41417-023-00637-8)

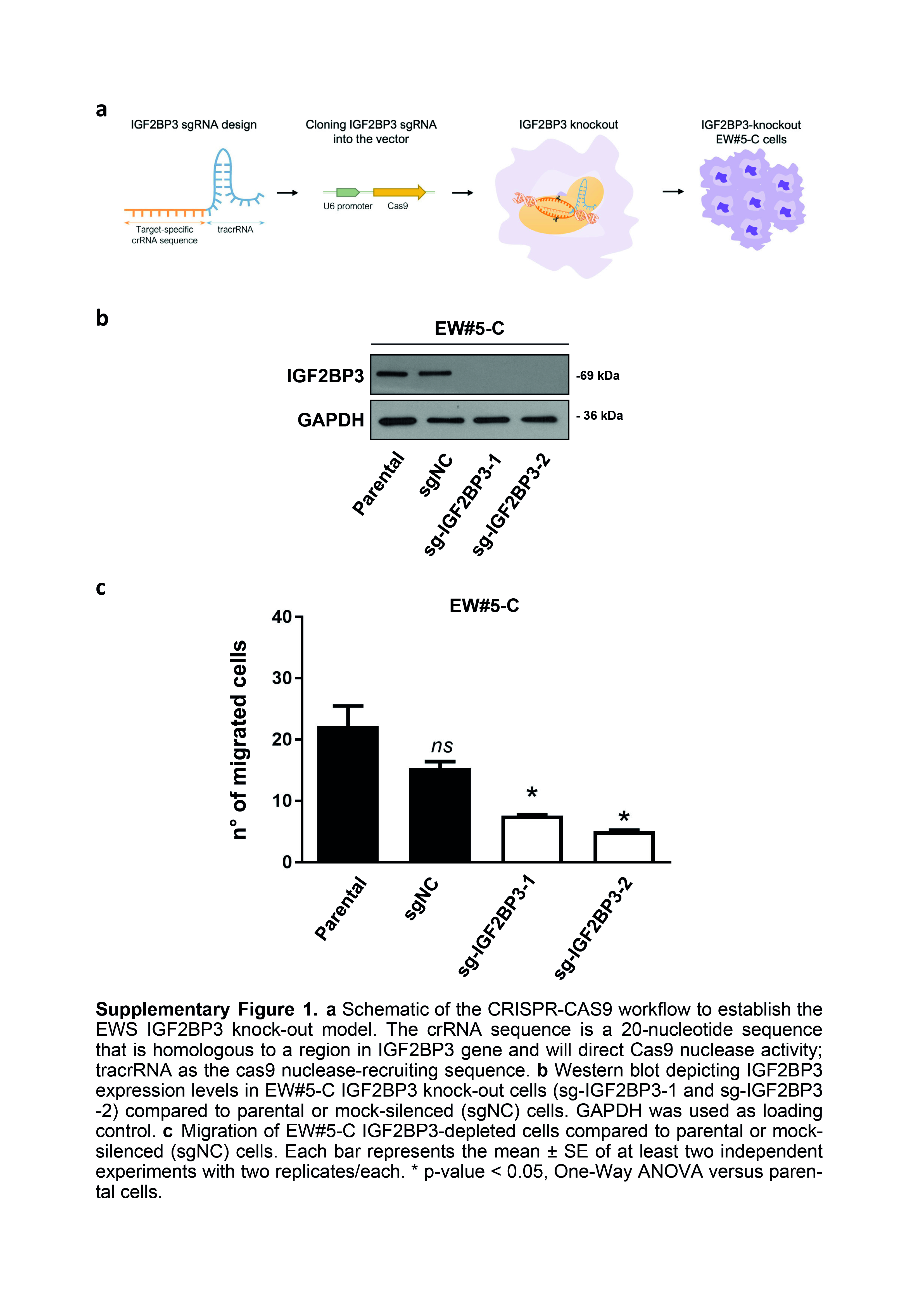

Supplement: Supplementary file 1 — Supplementary Figure 1 [file 41417_2023_637_MOESM1_ESM.tif]

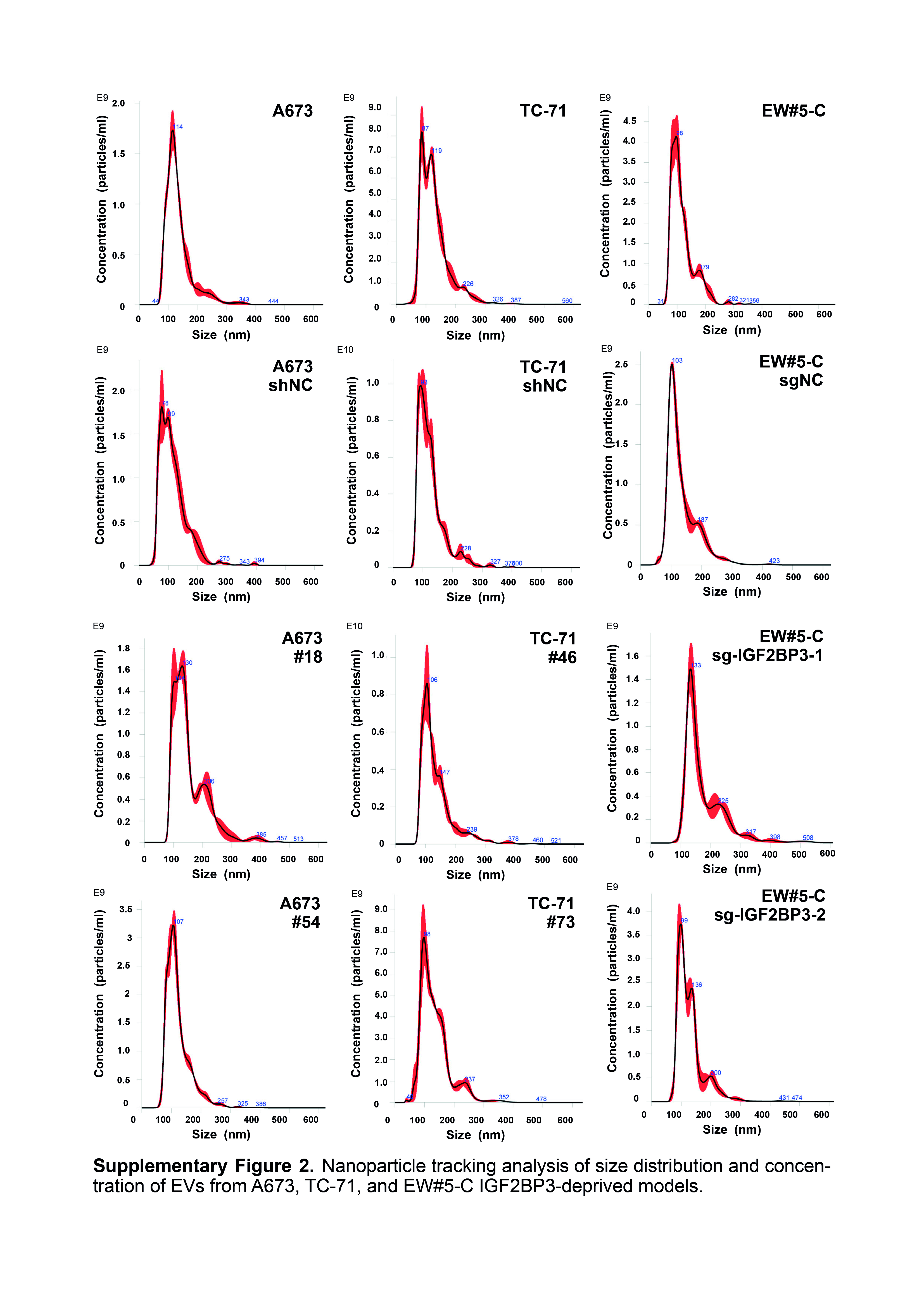

Supplement: Supplementary file 2 — Supplementary Figure 2 [file 41417_2023_637_MOESM2_ESM.tif]

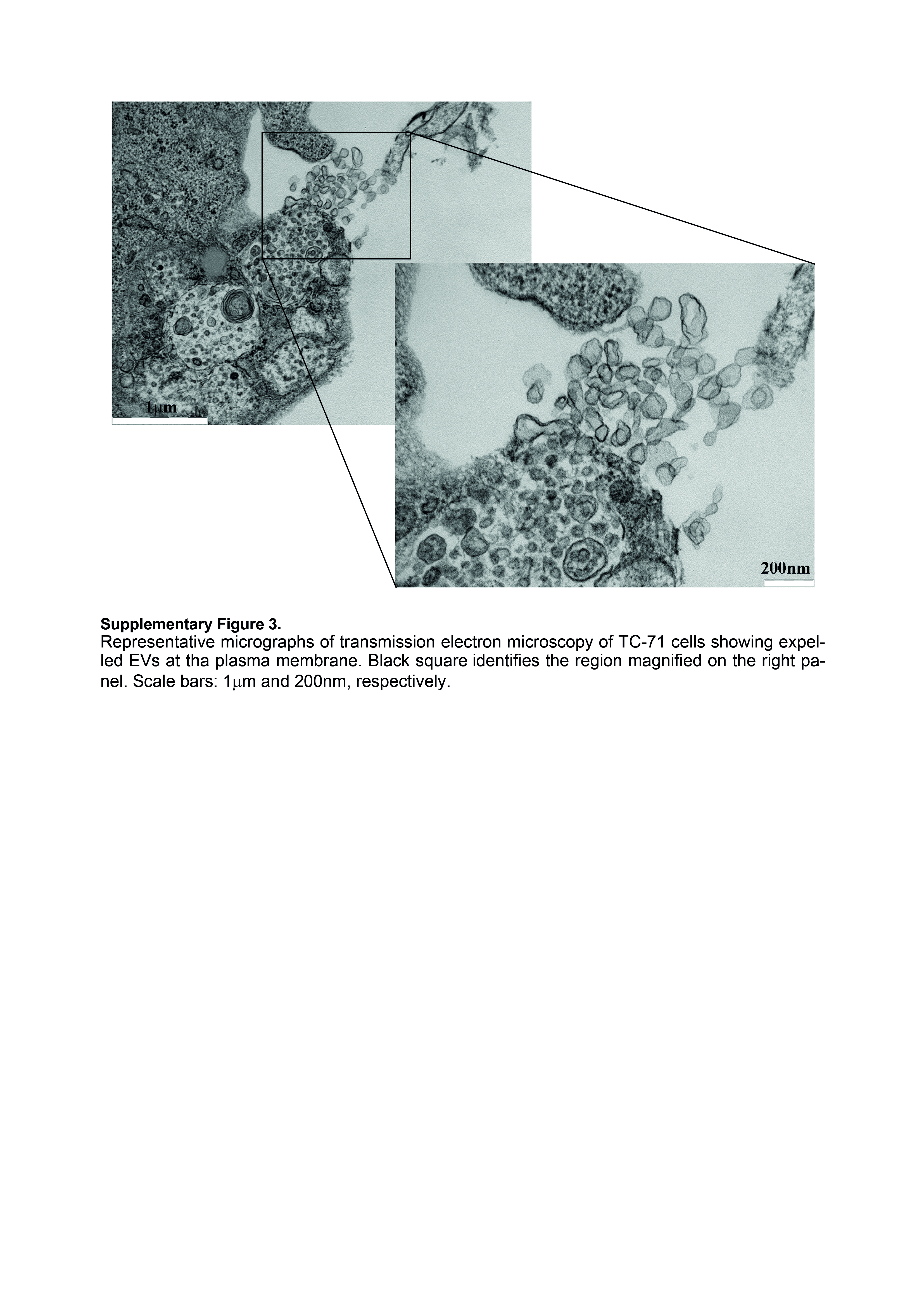

Supplement: Supplementary file 3 — Supplementary Figure 3 [file 41417_2023_637_MOESM3_ESM.tif]

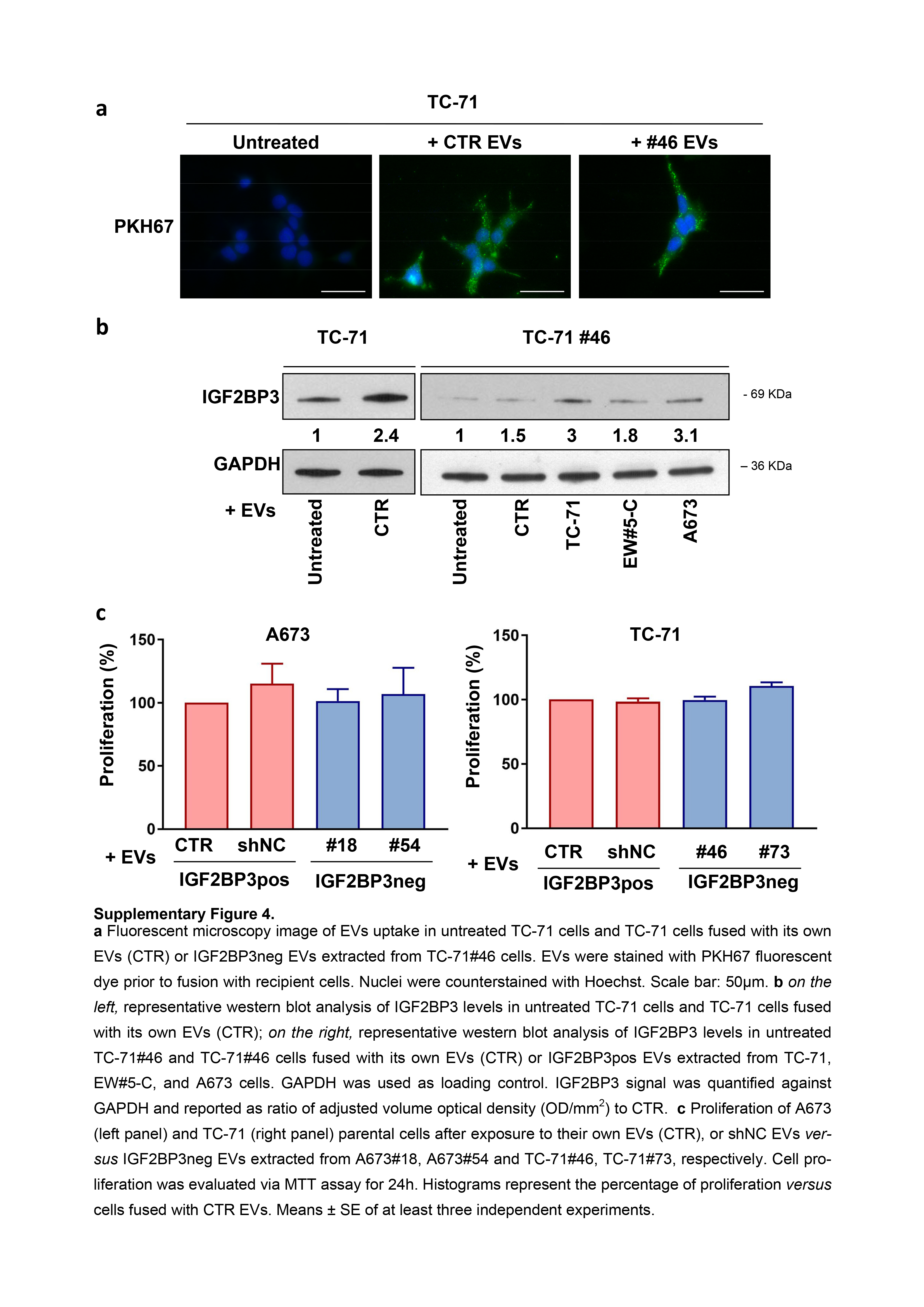

Supplement: Supplementary file 4 — Supplementary Figure 4 [file 41417_2023_637_MOESM4_ESM.tif]

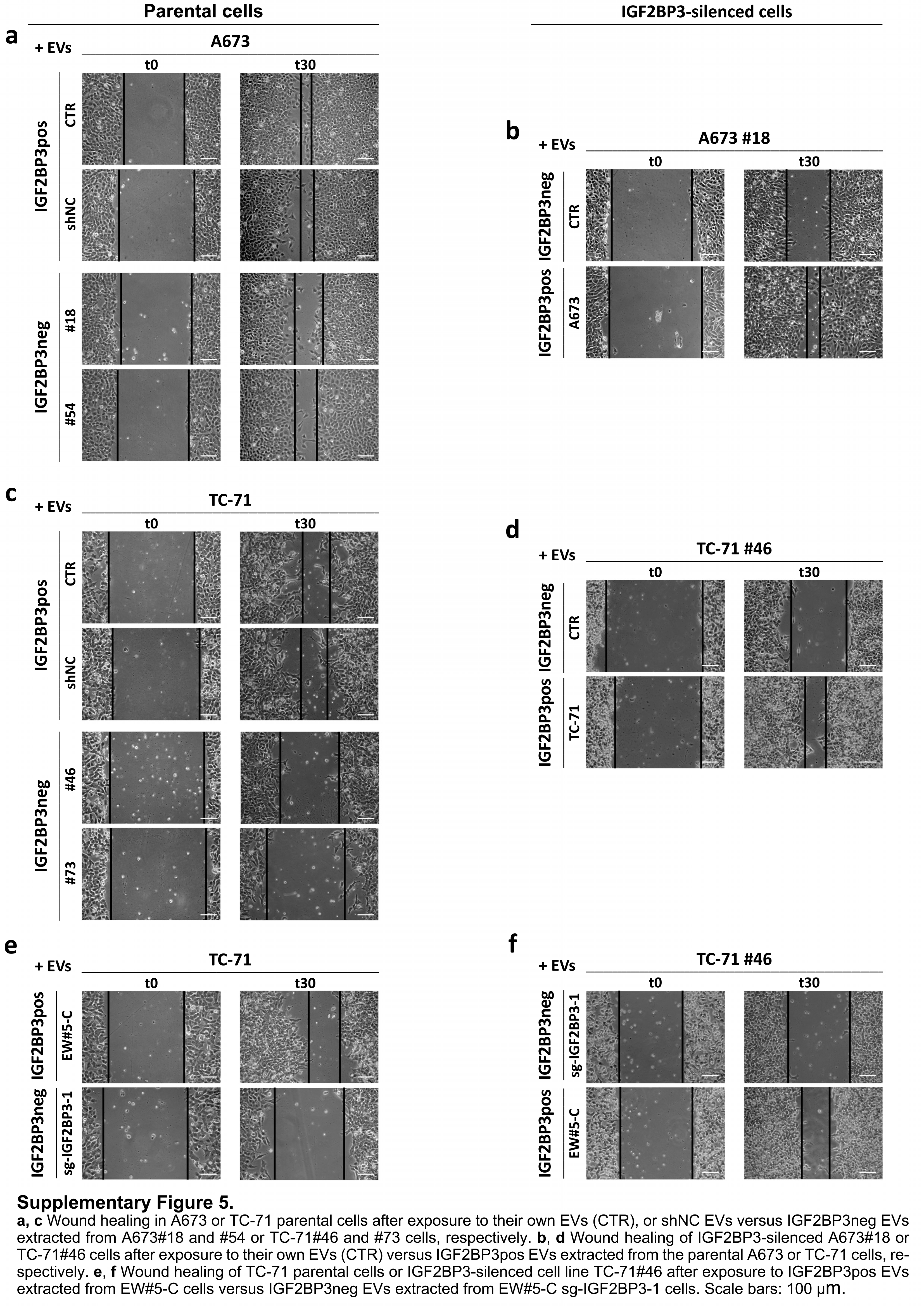

Supplement: Supplementary file 5 — Supplementary Figure 5 [file 41417_2023_637_MOESM5_ESM.tif]

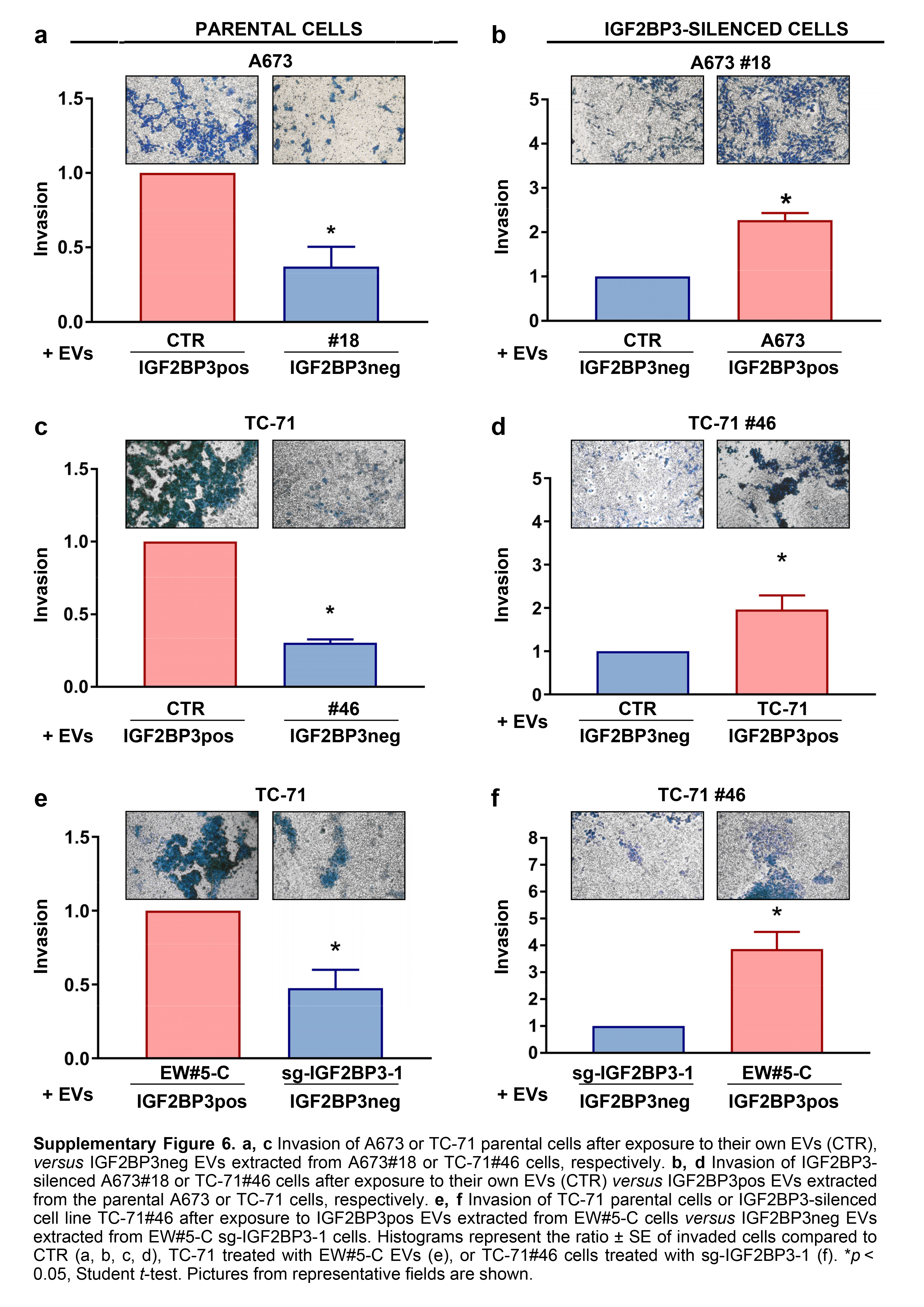

Supplement: Supplementary file 6 — Supplementary Figure 6 [file 41417_2023_637_MOESM6_ESM.tif]

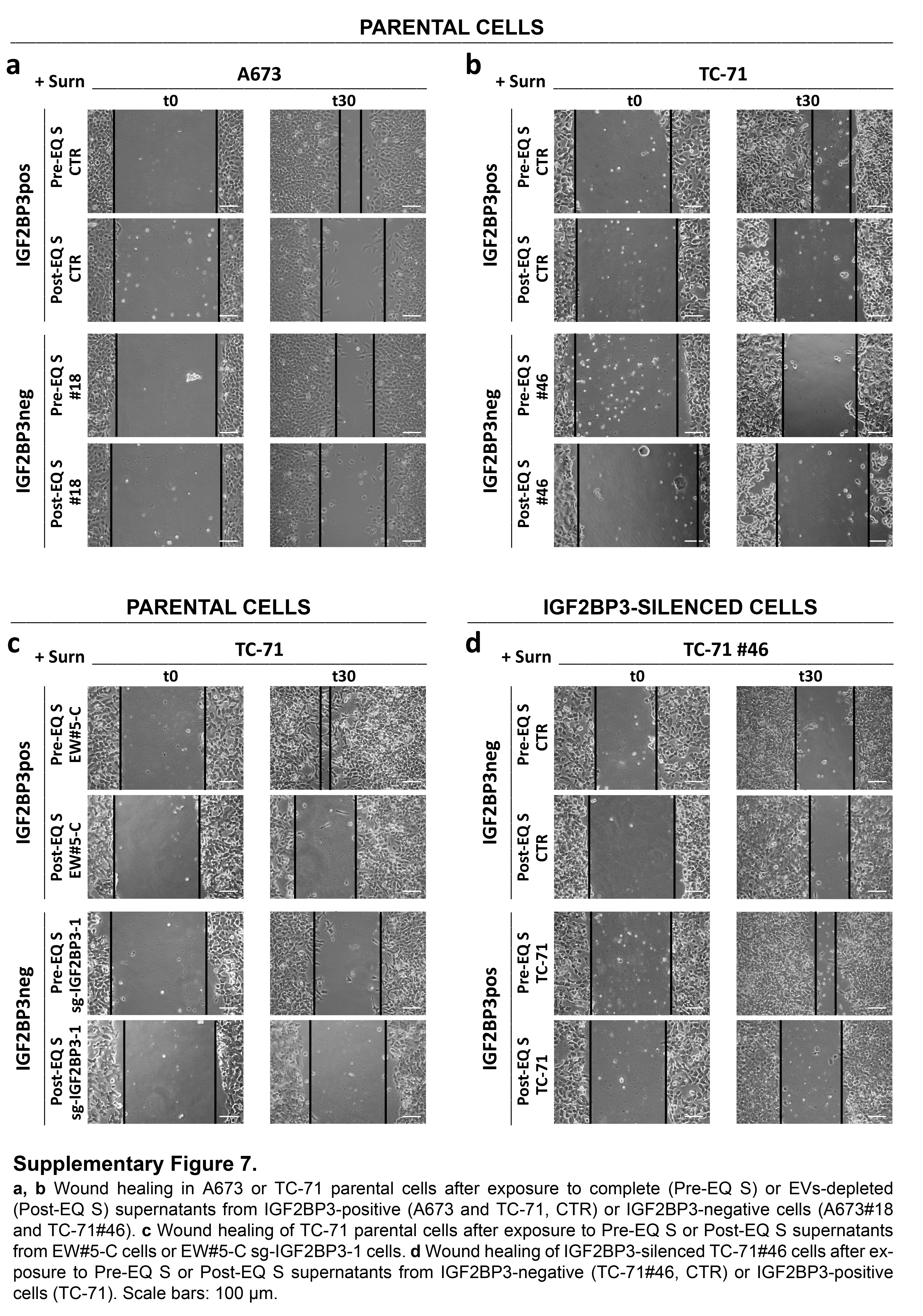

Supplement: Supplementary file 7 — Supplementary Figure 7 [file 41417_2023_637_MOESM7_ESM.tif]

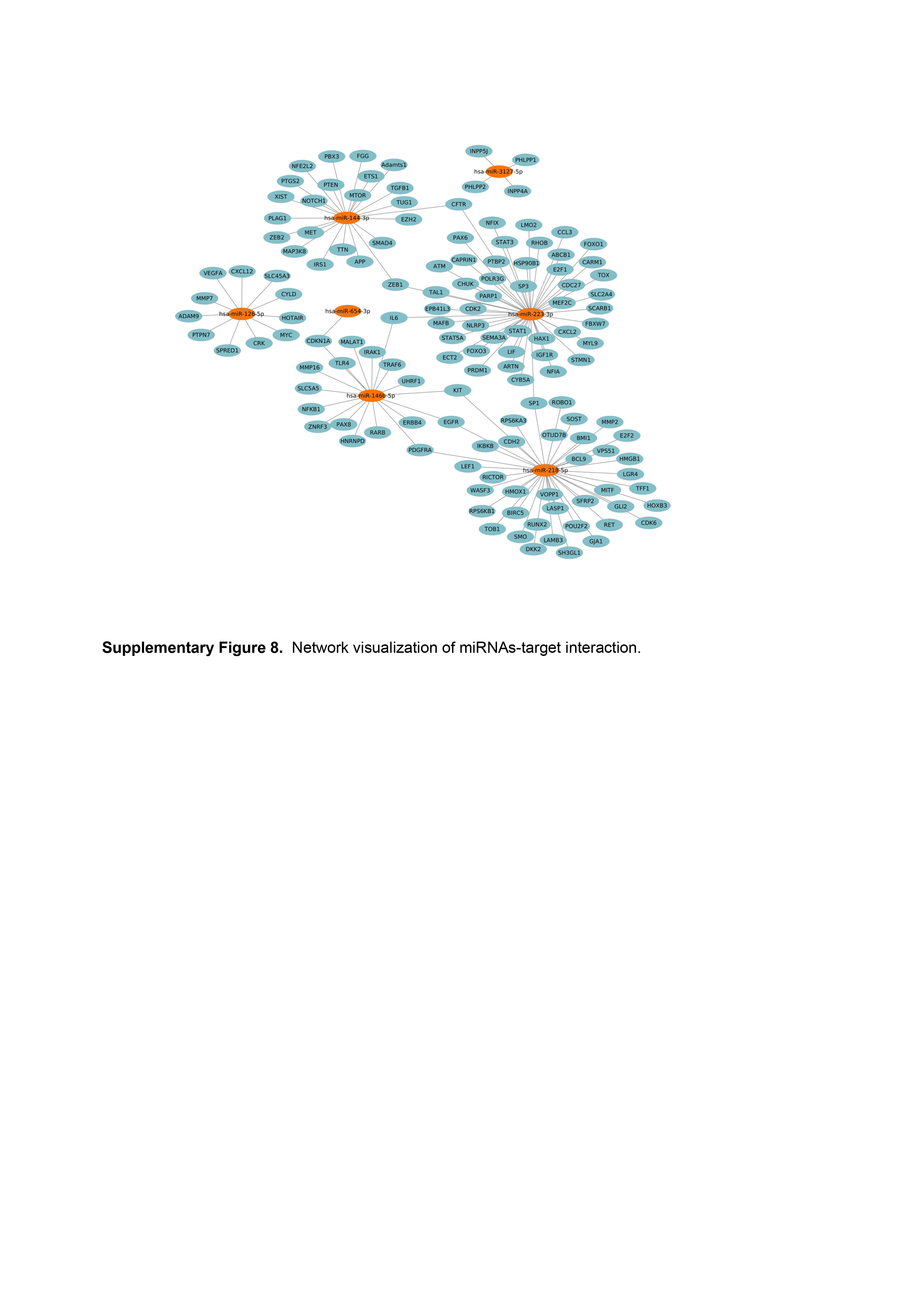

Supplement: Supplementary file 8 — Supplementary Figure 8 [file 41417_2023_637_MOESM8_ESM.tif]

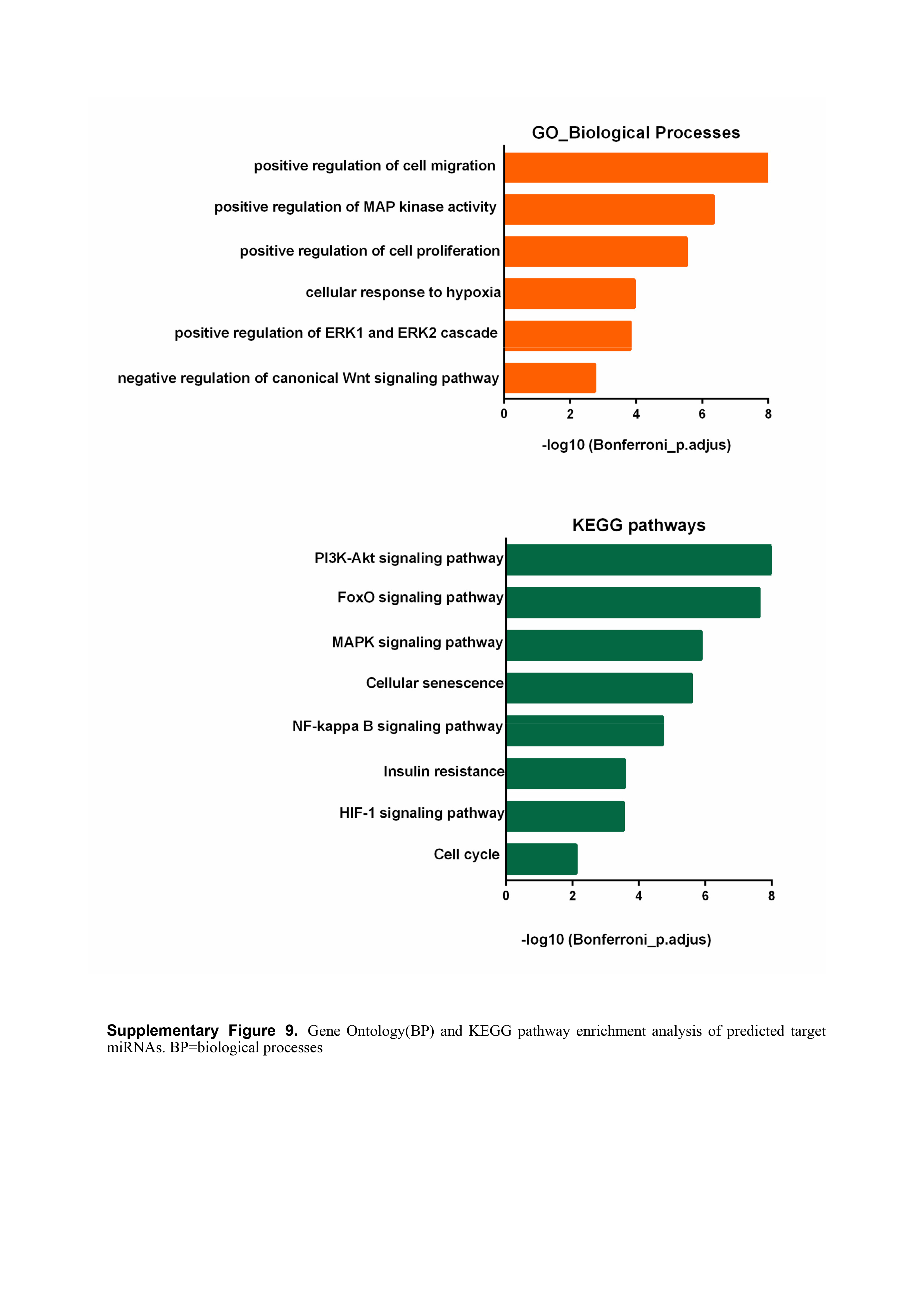

Supplement: Supplementary file 9 — Supplementary Figure 9 [file 41417_2023_637_MOESM9_ESM.tif]
