# Supplementary material for: Extracellular vesicle-associated IGF2BP3 tunes Ewing sarcoma cell migration and affects PI3K/Akt pathway in neighboring cells
Source: Cancer Gene Ther. 2023 Jun 23;30(9):1285–95. doi: 10.1038/s41417-023-00637-8 (PMC10501906; doi:10.1038/s41417-023-00637-8)
Supplement: Supplementary file 10 — Supplementary Table 1 [file 41417_2023_637_MOESM10_ESM.doc]

**Supplementary Table 1**: Size and concentration of EVs from IGF2BP3-depleted, parental or mock-silenced (shNC, sgNC) A673, TC-71 and EWS#5-C EWS cells analyzed by NanosightTM.

|  |  | |  |
| --- | --- | --- | --- |
| **EVs** | **Size (nm)** | | **Concentration (10^11^particles/ml)** |
| **A673** | 134.5 ± 4.2 | | 1.15 ± 0.0333 |
| **A673 shNC** | 116.8 ± 3.1 | | 1.52 ± 0.0922 |
| **A673 #18** | 155.0 ± 2.6 | | 1.50 ± 0.041 |
| **A673** **#54** | 122.6 ± 1.1 | | 2.09 ± 0.0572 |
| **TC-71** | 128.8 ± 3.6 | | 6.33 ± 0.0168 |
| **TC-71 shNC** | 126.2 ± 0.8 | | 6.95 ± 0.0309 |
| **TC-71 #46** | 137.0 ± 3.2 | | 5.72 ± 0.0362 |
| **TC-71 #73** | 135.6 ± 3.7 | | 5.89 ± 0.5960 |
| **EW#5-C** | 117.9 ± 1.8 | | 2.75 ± 0.0843 |
| **EW#5-C sgNC** | 135.9 ± 0.6 | | 1.58 ± 0.0258 |
| **EW#5-C sg-IGF2BP3-1** | 177.6 ± 2.3 | | 1.04 ± 0.0811 |
| **EW#5-C sg-IGF2BP3-2** | 129.9 ± 1.0 | | 2.36 ± 0.1310 |
|  | |  |  |
|  |  | |  |
