# Supplementary material for: Extracellular vesicle-associated IGF2BP3 tunes Ewing sarcoma cell migration and affects PI3K/Akt pathway in neighboring cells
Source: Cancer Gene Ther. 2023 Jun 23;30(9):1285–95. doi: 10.1038/s41417-023-00637-8 (PMC10501906; doi:10.1038/s41417-023-00637-8)
Supplement: Supplementary file 11 — Supplementary Table 2 [file 41417_2023_637_MOESM11_ESM.doc]

**Supplementary Table 2**: 73 and 40 differentially expressed miRNAs between parental/mock-silenced cells versus those derived from IGF2BP3 knock-down cells in A673 and TC-71 models, respectively. The miRNAs were sorted according to fold change.

| **A673 MODEL** | | |
| --- | --- | --- |
| ***miR_ID*** | ***log_2_FoldChange*** | ***P-value*** |
| hsa-miR-6781-5p | 4,291746207 | 0,02875607 |
| hsa-miR-124-3p | 4,239579354 | 0,003539558 |
| hsa-miR-10396a-3p | 4,232193823 | 0,018180316 |
| hsa-miR-450b-3p | 4,102491094 | 0,023323035 |
| hsa-miR-4453 | 3,724482278 | 0,031502002 |
| hsa-miR-3661 | 3,653262729 | 0,035826481 |
| hsa-miR-548q | 3,486007952 | 0,048832416 |
| hsa-miR-7107-5p | 3,276784077 | 0,036551372 |
| hsa-miR-4722-5p | 2,619279944 | 0,039809798 |
| hsa-miR-135b-5p | 1,622586013 | 0,013287579 |
| hsa-miR-9985 | 1,294496827 | 0,049038824 |
| hsa-miR-138-5p | 1,159116197 | 2,97026E-05 |
| hsa-miR-483-3p | 1,102830291 | 0,013616972 |
| hsa-miR-1226-3p | 1,075549923 | 0,012111498 |
| hsa-miR-141-3p | 0,956736148 | 0,03686418 |
| hsa-miR-144-3p | 0,925366125 | 0,019974077 |
| hsa-miR-146a-5p | 0,921749876 | 0,015524353 |
| hsa-miR-125b-2-3p | 0,804134943 | 0,039073169 |
| hsa-miR-20a-5p | 0,786407776 | 0,024931386 |
| hsa-miR-335-5p | 0,775489032 | 0,037656114 |
| hsa-miR-18a-5p | 0,738584354 | 0,034328177 |
| hsa-miR-142-3p | 0,721539424 | 0,04049424 |
| hsa-miR-1306-5p | 0,714619053 | 0,017394698 |
| hsa-miR-150-5p | 0,659323604 | 0,013085014 |
| hsa-miR-142-5p | 0,598167199 | 0,035321244 |
| hsa-miR-223-3p | 0,567074533 | 0,032703928 |
| hsa-miR-126-5p | 0,557893338 | 0,027274434 |
| hsa-miR-873-5p | 0,53630374 | 0,041270475 |
| hsa-miR-345-3p | 0,502028433 | 0,029068747 |
| hsa-miR-152-3p | 0,481509551 | 0,042070661 |
| hsa-miR-151a-3p | -0,502831133 | 0,050520601 |
| hsa-miR-95-3p | -0,54435513 | 0,022099659 |
| hsa-miR-431-5p | -0,595434516 | 0,039416457 |
| hsa-miR-132-3p | -0,617388081 | 0,028389336 |
| hsa-miR-487b-3p | -0,635829131 | 0,014018344 |
| hsa-miR-532-5p | -0,640480473 | 0,013459869 |
| hsa-miR-30a-5p | -0,654253277 | 0,025978022 |
| hsa-miR-501-3p | -0,667511605 | 0,031242456 |
| hsa-miR-221-3p | -0,761241311 | 0,002740307 |
| hsa-miR-3663-5p | -0,762746866 | 0,038635641 |
| hsa-miR-146b-5p | -0,780169966 | 0,009238718 |
| hsa-miR-99b-5p | -0,78501677 | 0,000854284 |
| hsa-miR-889-3p | -0,806859084 | 0,04083997 |
| hsa-miR-500a-3p | -0,881912799 | 0,019532796 |
| hsa-miR-134-5p | -0,912275513 | 0,004900436 |
| hsa-miR-370-3p | -0,923138074 | 0,018955679 |
| hsa-miR-329-3p | -0,932375169 | 0,007505044 |
| hsa-miR-125b-1-3p | -0,968953026 | 0,012721365 |
| hsa-miR-339-5p | -0,994806833 | 0,004560092 |
| hsa-miR-628-5p | -1,002856265 | 0,007151849 |
| hsa-miR-218-5p | -1,005275513 | 0,00014704 |
| hsa-let-7e-5p | -1,038325698 | 0,004061952 |
| hsa-miR-654-3p | -1,063382659 | 0,003297335 |
| hsa-miR-222-3p | -1,089345091 | 4,79715E-05 |
| hsa-miR-769-3p | -1,133790674 | 0,047468056 |
| hsa-miR-504-5p | -1,221340678 | 0,002600991 |
| hsa-miR-3127-5p | -1,308739647 | 0,023228293 |
| hsa-miR-221-5p | -1,364105255 | 0,000891393 |
| hsa-miR-196a-5p | -1,428982546 | 0,000609076 |
| hsa-miR-12136 | -1,440380068 | 0,000306449 |
| hsa-miR-137-3p | -1,486561807 | 0,000680848 |
| hsa-miR-5001-3p | -1,523238153 | 0,024019254 |
| hsa-miR-329-5p | -1,563776958 | 0,044247539 |
| hsa-miR-3125 | -1,612087535 | 0,028169833 |
| hsa-miR-664a-5p | -1,646141601 | 0,00267726 |
| hsa-miR-3065-3p | -1,714353516 | 0,019050292 |
| hsa-miR-3129-5p | -1,72440535 | 0,005785323 |
| hsa-miR-10395-3p | -1,750111644 | 0,026174937 |
| hsa-miR-892c-3p | -2,517847405 | 0,030873393 |
| hsa-miR-3158-3p | -2,628649347 | 0,045064041 |
| hsa-miR-548e-3p | -2,784419261 | 0,006272119 |
| hsa-miR-495-5p | -3,30448612 | 0,035379399 |
| hsa-miR-3129-3p | -3,764277948 | 0,005129595 |
|  |  |  |
|  |  |  |
|  |  |  |
|  |  |  |
| **TC-71 MODEL** | | |
| ***miR_ID*** | ***log_2_FoldChange*** | ***P-value*** |
| hsa-miR-6796-5p | 4,25103853 | 0,020430624 |
| hsa-miR-6875-5p | 4,19573409 | 0,012830245 |
| hsa-miR-6794-3p | 3,409847053 | 0,032217668 |
| hsa-miR-30c-2-3p | 2,631615938 | 0,043180545 |
| hsa-let-7f-2-3p | 2,445700633 | 0,01535182 |
| hsa-miR-449a | 1,582193162 | 0,035414361 |
| hsa-miR-504-5p | 1,074640146 | 0,035745236 |
| hsa-miR-181a-2-3p | 0,942684372 | 0,004420998 |
| hsa-miR-1306-5p | 0,754184732 | 0,020171967 |
| hsa-miR-34a-5p | 0,692755963 | 0,014172064 |
| hsa-miR-199b-5p | 0,674880671 | 0,010175393 |
| hsa-miR-144-3p | 0,659812389 | 0,001151058 |
| hsa-miR-195-5p | 0,521877739 | 0,018084547 |
| hsa-miR-223-3p | 0,509571715 | 0,005702666 |
| hsa-miR-126-5p | 0,496261604 | 0,002762947 |
| hsa-miR-654-3p | -0,557988204 | 0,034593675 |
| hsa-miR-218-5p | -0,604104897 | 0,005354698 |
| hsa-miR-378a-3p | -0,605554157 | 7,38723E-05 |
| hsa-miR-941 | -0,643566975 | 0,033389404 |
| hsa-miR-378c | -0,661234013 | 0,007091166 |
| hsa-miR-320e | -0,717817205 | 0,009355662 |
| hsa-miR-4485-3p | -0,734648328 | 0,02828542 |
| hsa-miR-146b-5p | -0,779530977 | 8,70574E-05 |
| hsa-miR-490-5p | -0,816784976 | 0,002680184 |
| hsa-miR-326 | -0,862430166 | 0,006469925 |
| hsa-miR-12136 | -0,875789454 | 0,033983012 |
| hsa-miR-1246 | -1,033849243 | 0,028569349 |
| hsa-miR-10527-5p | -1,070366505 | 0,037152065 |
| hsa-miR-337-5p | -1,092543718 | 0,004328687 |
| hsa-miR-378i | -1,101339642 | 0,019692093 |
| hsa-miR-598-3p | -1,103704454 | 0,000679111 |
| hsa-miR-664a-5p | -1,191129978 | 0,03075797 |
| hsa-miR-16-2-3p | -1,211389244 | 0,00924588 |
| hsa-miR-3177-3p | -1,617440655 | 0,013303554 |
| hsa-miR-3115 | -1,655679522 | 0,017459487 |
| hsa-miR-3127-5p | -1,6815829 | 0,000758874 |
| hsa-miR-6514-5p | -2,218983332 | 0,028764816 |
| hsa-miR-5697 | -3,495894063 | 0,019642752 |
| hsa-miR-101-2-5p | -3,794003057 | 0,036086603 |
| hsa-miR-3167 | -4,491085184 | 0,00215652 |
