# Supplementary material for: Extracellular vesicle-associated IGF2BP3 tunes Ewing sarcoma cell migration and affects PI3K/Akt pathway in neighboring cells
Source: Cancer Gene Ther. 2023 Jun 23;30(9):1285–95. doi: 10.1038/s41417-023-00637-8 (PMC10501906; doi:10.1038/s41417-023-00637-8)
Supplement: Supplementary file 12 — Supplementary Table 3 [file 41417_2023_637_MOESM12_ESM.doc]

| **Supplementary Table 3:** GO biological processes | | |  |  |  |  |  |  |  |  |  |
| --- | --- | --- | --- | --- | --- | --- | --- | --- | --- | --- | --- |
| **Term** | **Count** | **%** | **PValue** | **Genes** | **List Total** | **Pop Hits** | **Pop Total** | **Bonferroni** | **Benjamini** | **FDR** | **Fold Enrichment** |
| GO:0030335~positive regulation of cell migration | 18 | 13,1386861 | 2,32E-12 | RET, PDGFRA, TGFB1, NOTCH1, MMP7, SEMA3A, MMP2, LEF1, STAT3, EGFR, IGF1R, VEGFA, CXCL12, SMO, KIT, CCL3, ADAM9, ATM | 134 | 256 | 19256 | 5,5588E-09 | 9,2646E-10 | 8,4603E-10 | 10,10401119 |
| GO:0043406~positive regulation of MAP kinase activity | 11 | 8,02919708 | 1,9175E-10 | APP, MEF2C, TGFB1, IRAK1, KIT, ADAM9, TLR4, EGFR, EZH2, ROBO1, VEGFA | 134 | 80 | 19256 | 4,5943E-07 | 6,5633E-08 | 5,9935E-08 | 19,75895522 |
| GO:0008284~positive regulation of cell proliferation | 21 | 15,3284672 | 1,2271E-09 | PDGFRA, TGFB1, NOTCH1, IRS1, LEF1, PTEN, LIF, ETS1, EGFR, RUNX2, IGF1R, VEGFA, IL6, SFRP2, ERBB4, MYC, CARM1, KIT, CDK2, BIRC5, EZH2 | 134 | 549 | 19256 | 2,94E-06 | 3,2667E-07 | 2,9831E-07 | 5,496778403 |
| GO:0071456~cellular response to hypoxia | 11 | 8,02919708 | 4,7165E-08 | IRAK1, MYC, PTEN, E2F1, HMOX1, MALAT1, SLC2A4, FOXO3, PTGS2, MTOR, VEGFA | 134 | 140 | 19256 | 0,000113 | 7,063E-06 | 6,4498E-06 | 11,29083156 |
| GO:0070374~positive regulation of ERK1 and ERK2 cascade | 13 | 9,48905109 | 6,1829E-08 | PDGFRA, APP, TGFB1, NOTCH1, FBXW7, FGG, PTEN, HMGB1, EGFR, VEGFA, ERBB4, CCL3, TLR4 | 134 | 228 | 19256 | 0,00014813 | 8,6337E-06 | 7,8842E-06 | 8,193506153 |
| GO:0090090~negative regulation of canonical Wnt signaling pathway | 10 | 7,29927007 | 7,4749E-07 | CYLD, APP, NOTCH1, ZNRF3, SFRP2, CDH2, SOST, FOXO3, DKK2, FOXO1 | 134 | 145 | 19256 | 0,00178938 | 8,1408E-05 | 7,4341E-05 | 9,910447761 |
| GO:0006915~apoptotic process | 17 | 12,4087591 | 4,5964E-06 | MEF2C, PHLPP1, PARP1, SEMA3A, PTEN, FOXO1, NFKB1, RHOB, RPS6KA3, GJA1, SFRP2, SMO, RPS6KB1, EPB41L3, BIRC5, NLRP3, RARB | 134 | 607 | 19256 | 0,01095248 | 0,0003671 | 0,00033523 | 4,024588753 |
