# Supplementary material for: Extracellular vesicle-associated IGF2BP3 tunes Ewing sarcoma cell migration and affects PI3K/Akt pathway in neighboring cells
Source: Cancer Gene Ther. 2023 Jun 23;30(9):1285–95. doi: 10.1038/s41417-023-00637-8 (PMC10501906; doi:10.1038/s41417-023-00637-8)
Supplement: Supplementary file 13 — Supplementary Table 4 [file 41417_2023_637_MOESM13_ESM.doc]

**Supplementary Table 4:** KEGG_PATHWAY

| **Term** | **Count** | **%** | **PValue** | **Genes** | **List Total** | **Pop Hits** | **Pop Total** | **Bonferroni** | **Benjamini** | **FDR** | **Fold Enrichment** |
| --- | --- | --- | --- | --- | --- | --- | --- | --- | --- | --- | --- |
| hsa05200:Pathways in cancer | 45 | 32,8467153 | 5,2486E-25 | RET, CDKN1A, NOTCH1, LEF1, PTEN, PTGS2, ETS1, FOXO1, EGFR, HSP90B1, IGF1R, GLI2, IKBKB, MYC, E2F1, E2F2, HMOX1, STAT5A, PDGFRA, SMAD4, TGFB1, LAMB3, CHUK, STAT1, MMP2, STAT3, MITF, MTOR, NFKB1, VEGFA, IL6, CXCL12, CDK6, SMO, PAX8, RPS6KB1, SP1, TRAF6, KIT, CDK2, BIRC5, RARB, MET, CRK, NFE2L2 | 108 | 531 | 8156 | 1,0497E-22 | 1,0497E-22 | 5,4586E-23 | 6,399874451 |
| hsa05206:MicroRNAs in cancer | 28 | 20,4379562 | 1,4567E-15 | CDKN1A, SLC45A3, NOTCH1, ABCB1, IRS1, PTEN, BMI1, PTGS2, EGFR, IKBKB, MYC, STMN1, E2F1, E2F2, HMOX1, PDGFRA, STAT3, MTOR, NFKB1, VEGFA, ZEB2, ZEB1, CDK6, MMP16, ATM, MET, CRK, EZH2 | 108 | 310 | 8156 | 2,8866E-13 | 1,4567E-13 | 7,5749E-14 | 6,821027479 |
| hsa04151:PI3K-Akt signaling pathway | 25 | 18,2481752 | 1,7127E-11 | PHLPP2, CDKN1A, PHLPP1, IRS1, PTEN, FOXO3, EGFR, HSP90B1, IGF1R, IKBKB, ERBB4, MYC, PDGFRA, LAMB3, CHUK, MTOR, NFKB1, VEGFA, IL6, CDK6, RPS6KB1, KIT, CDK2, MET, TLR4 | 108 | 354 | 8156 | 3,4254E-09 | 6,8508E-10 | 3,5624E-10 | 5,333228709 |
| hsa04068:FoxO signaling pathway | 16 | 11,6788321 | 1,1733E-10 | CDKN1A, SMAD4, TGFB1, CHUK, IRS1, STAT3, PTEN, SLC2A4, FOXO3, FOXO1, EGFR, IGF1R, IKBKB, IL6, CDK2, ATM | 108 | 131 | 8156 | 2,3466E-08 | 2,9333E-09 | 1,5253E-09 | 9,22363585 |
| hsa04010:MAPK signaling pathway | 20 | 14,5985401 | 6,5809E-09 | PDGFRA, MEF2C, TGFB1, CHUK, EGFR, NFKB1, IGF1R, VEGFA, IKBKB, RPS6KA3, IRAK1, ERBB4, MYC, TRAF6, KIT, STMN1, MAP3K8, PTPN7, MET, CRK | 108 | 294 | 8156 | 1,3162E-06 | 1,0125E-07 | 5,2647E-08 | 5,137314185 |
| hsa04218:Cellular senescence | 15 | 10,9489051 | 1,2823E-08 | CDKN1A, TGFB1, PTEN, FOXO3, ETS1, FOXO1, MTOR, NFKB1, IL6, CDK6, MYC, CDK2, E2F1, E2F2, ATM | 108 | 156 | 8156 | 2,5646E-06 | 1,8318E-07 | 9,5255E-08 | 7,261396011 |
| hsa04064:NF-kappa B signaling pathway | 12 | 8,75912409 | 9,5053E-08 | IKBKB, CYLD, CXCL12, IRAK1, CHUK, PARP1, TRAF6, ATM, PTGS2, CXCL2, TLR4, NFKB1 | 108 | 104 | 8156 | 1,901E-05 | 9,0527E-07 | 4,7074E-07 | 8,713675214 |
| hsa05235:PD-L1 expression and PD-1 checkpoint pathway in cancer | 11 | 8,02919708 | 2,1173E-07 | IKBKB, RPS6KB1, CHUK, STAT1, TRAF6, STAT3, PTEN, TLR4, EGFR, NFKB1, MTOR | 108 | 89 | 8156 | 4,2345E-05 | 1,7644E-06 | 9,1749E-07 | 9,33374948 |
| hsa04931:Insulin resistance | 11 | 8,02919708 | 1,3108E-06 | IKBKB, RPS6KA3, IL6, RPS6KB1, IRS1, STAT3, PTEN, SLC2A4, FOXO1, NFKB1, MTOR | 108 | 108 | 8156 | 0,00026213 | 9,3628E-06 | 4,8687E-06 | 7,69170096 |
| hsa04066:HIF-1 signaling pathway | 11 | 8,02919708 | 1,4276E-06 | IL6, CDKN1A, RPS6KB1, STAT3, HMOX1, TLR4, EGFR, NFKB1, MTOR, IGF1R, VEGFA | 108 | 109 | 8156 | 0,00028547 | 9,8452E-06 | 5,1195E-06 | 7,621134896 |
| hsa04110:Cell cycle | 10 | 7,29927007 | 3,7731E-05 | CDKN1A, SMAD4, TGFB1, CDK6, MYC, CDC27, CDK2, E2F1, E2F2, ATM | 108 | 126 | 8156 | 0,00751791 | 0,00016769 | 8,72E-05 | 5,993533216 |
