# Supplementary material for: Extracellular vesicle-associated IGF2BP3 tunes Ewing sarcoma cell migration and affects PI3K/Akt pathway in neighboring cells
Source: Cancer Gene Ther. 2023 Jun 23;30(9):1285–95. doi: 10.1038/s41417-023-00637-8 (PMC10501906; doi:10.1038/s41417-023-00637-8)
Supplement: Supplementary file 14 — Supplementary Table 5 [file 41417_2023_637_MOESM14_ESM.doc]

**Supplementary Table 5**: miRNA IDs included in Agilent Human miRNA Microarray v.2 and the corresponding miRNA IDs in RNAseq.

| **ARRAY_miRNAID** | **RNA_SEQ_miRNAID** |  |
| --- | --- | --- |
| hsa-let-7a | hsa-let-7a-5p |  |
| hsa-let-7a* | hsa-let-7a-3p |  |
| hsa-let-7b | hsa-let-7b-5p |  |
| hsa-let-7b* | hsa-let-7b-3p |  |
| hsa-let-7c | hsa-let-7c-5p |  |
| hsa-let-7c* | hsa-let-7c-3p |  |
| hsa-let-7d | hsa-let-7d-5p |  |
| hsa-let-7d* | hsa-let-7d-3p |  |
| hsa-let-7e | hsa-let-7e-5p |  |
| hsa-let-7f | hsa-let-7f-5p |  |
| hsa-let-7f-1* | hsa-let-7f-1-3p |  |
| hsa-let-7g | hsa-let-7g-5p |  |
| hsa-let-7i | hsa-let-7i-5p |  |
| hsa-let-7i* | hsa-let-7i-3p |  |
| hsa-miR-1 | hsa-miR-1-3p |  |
| hsa-miR-100 | hsa-miR-100-5p |  |
| hsa-miR-101 | hsa-miR-101-3p |  |
| hsa-miR-103 | hsa-miR-103a-3p |  |
| hsa-miR-105 | hsa-miR-105-5p |  |
| hsa-miR-106a* | hsa-miR-106a-3p |  |
| hsa-miR-106b | hsa-miR-106b-5p |  |
| hsa-miR-106b* | hsa-miR-106b-3p |  |
| hsa-miR-107 | hsa-miR-107 |  |
| hsa-miR-10a | hsa-miR-10a-5p |  |
| hsa-miR-10b | hsa-miR-10b-5p |  |
| hsa-miR-10b* | hsa-miR-10b-3p |  |
| hsa-miR-122 | hsa-miR-122-5p |  |
| hsa-miR-1224-5p | hsa-miR-1224-5p |  |
| hsa-miR-1225-5p | hsa-miR-1225-5p |  |
| hsa-miR-1226* | hsa-miR-1226-5p |  |
| hsa-miR-1228 | hsa-miR-1228-3p |  |
| hsa-miR-1233 | hsa-miR-1233 |  |
| hsa-miR-1234 | hsa-miR-1234 |  |
| hsa-miR-1236 | hsa-miR-1236 |  |
| hsa-miR-1238 | hsa-miR-1238 |  |
| hsa-miR-124 | hsa-miR-124-3p |  |
| hsa-miR-125a-3p | hsa-miR-125a-3p |  |
| hsa-miR-125a-5p | hsa-miR-125a-5p |  |
| hsa-miR-125b | hsa-miR-125b-5p |  |
| hsa-miR-125b-1* | hsa-miR-125b-1-3p |  |
| hsa-miR-125b-2* | hsa-miR-125b-2-3p |  |
| hsa-miR-126 | hsa-miR-126-3p |  |
| hsa-miR-126* | hsa-miR-126-5p |  |
| hsa-miR-127-3p | hsa-miR-127-3p |  |
| hsa-miR-128 | hsa-miR-128-3p |  |
| hsa-miR-129-5p | hsa-miR-129-5p |  |
| hsa-miR-129* | hsa-miR-129-1-3p |  |
| hsa-miR-130a | hsa-miR-130a-3p |  |
| hsa-miR-130a* | hsa-miR-130a-5p |  |
| hsa-miR-130b | hsa-miR-130b-3p |  |
| hsa-miR-132 | hsa-miR-132-3p |  |
| hsa-miR-132* | hsa-miR-132-5p |  |
| hsa-miR-133a | hsa-miR-133a-3p |  |
| hsa-miR-133b | hsa-miR-133b |  |
| hsa-miR-134 | hsa-miR-134 |  |
| hsa-miR-135a | hsa-miR-135a-5p |  |
| hsa-miR-135a* | hsa-miR-135a-3p |  |
| hsa-miR-135b | hsa-miR-135b-5p |  |
| hsa-miR-135b* | hsa-miR-135b-3p |  |
| hsa-miR-136 | hsa-miR-136-5p |  |
| hsa-miR-137 | hsa-miR-137 |  |
| hsa-miR-138 | hsa-miR-138-5p |  |
| hsa-miR-138-1* | hsa-miR-138-1-3p |  |
| hsa-miR-138-2* | hsa-miR-138-2-3p |  |
| hsa-miR-139-3p | hsa-miR-139-3p |  |
| hsa-miR-139-5p | hsa-miR-139-5p |  |
| hsa-miR-140-3p | hsa-miR-140-3p |  |
| hsa-miR-140-5p | hsa-miR-140-5p |  |
| hsa-miR-141 | hsa-miR-141-3p |  |
| hsa-miR-141* | hsa-miR-141-5p |  |
| hsa-miR-142-3p | hsa-miR-142-3p |  |
| hsa-miR-142-5p | hsa-miR-142-5p |  |
| hsa-miR-143 | hsa-miR-143-3p |  |
| hsa-miR-144 | hsa-miR-144-3p |  |
| hsa-miR-144* | hsa-miR-144-5p |  |
| hsa-miR-145 | hsa-miR-145-5p |  |
| hsa-miR-145* | hsa-miR-145-3p |  |
| hsa-miR-146a | hsa-miR-146a-5p |  |
| hsa-miR-146b-5p | hsa-miR-146b-5p |  |
| hsa-miR-147 | hsa-miR-147a |  |
| hsa-miR-148a | hsa-miR-148a-3p |  |
| hsa-miR-148a* | hsa-miR-148a-5p |  |
| hsa-miR-148b | hsa-miR-148b-3p |  |
| hsa-miR-149 | hsa-miR-149-5p |  |
| hsa-miR-149* | hsa-miR-149-3p |  |
| hsa-miR-150 | hsa-miR-150-5p |  |
| hsa-miR-150* | hsa-miR-150-3p |  |
| hsa-miR-151-3p | hsa-miR-151a-3p |  |
| hsa-miR-151-5p | hsa-miR-151a-5p |  |
| hsa-miR-152 | hsa-miR-152-3p |  |
| hsa-miR-153 | hsa-miR-153 |  |
| hsa-miR-154 | hsa-miR-154-5p |  |
| hsa-miR-155 | hsa-miR-155-5p |  |
| hsa-miR-15a | hsa-miR-15a-5p |  |
| hsa-miR-15a* | hsa-miR-15a-3p |  |
| hsa-miR-15b | hsa-miR-15b-5p |  |
| hsa-miR-15b* | hsa-miR-15b-3p |  |
| hsa-miR-16 | hsa-miR-16-5p |  |
| hsa-miR-16-1* | hsa-miR-16-1-3p |  |
| hsa-miR-16-2* | hsa-miR-16-2-3p |  |
| hsa-miR-17 | hsa-miR-17-5p |  |
| hsa-miR-17* | hsa-miR-17-3p |  |
| hsa-miR-181a | hsa-miR-181a-5p |  |
| hsa-miR-181a-2* | hsa-miR-181a-2-3p |  |
| hsa-miR-181a* | hsa-miR-181a-3p |  |
| hsa-miR-181b | hsa-miR-181b-5p |  |
| hsa-miR-181c | hsa-miR-181c-5p |  |
| hsa-miR-181c* | hsa-miR-181c-3p |  |
| hsa-miR-181d | hsa-miR-181d |  |
| hsa-miR-182 | hsa-miR-182-5p |  |
| hsa-miR-182* | hsa-miR-182-3p |  |
| hsa-miR-183 | hsa-miR-183-5p |  |
| hsa-miR-184 | hsa-miR-184 |  |
| hsa-miR-185 | hsa-miR-185-5p |  |
| hsa-miR-186 | hsa-miR-186-5p |  |
| hsa-miR-187 | hsa-miR-187-3p |  |
| hsa-miR-187* | hsa-miR-187-5p |  |
| hsa-miR-188-3p | hsa-miR-188-3p |  |
| hsa-miR-188-5p | hsa-miR-188-5p |  |
| hsa-miR-18a | hsa-miR-18a-5p |  |
| hsa-miR-18a* | hsa-miR-18a-3p |  |
| hsa-miR-18b | hsa-miR-18b-5p |  |
| hsa-miR-190 | hsa-miR-190a-5p |  |
| hsa-miR-190b | hsa-miR-190b |  |
| hsa-miR-191 | hsa-miR-191-5p |  |
| hsa-miR-191* | hsa-miR-191-3p |  |
| hsa-miR-192 | hsa-miR-192-5p |  |
| hsa-miR-192* | hsa-miR-192-3p |  |
| hsa-miR-193a-3p | hsa-miR-193a-3p |  |
| hsa-miR-193a-5p | hsa-miR-193a-5p |  |
| hsa-miR-193b | hsa-miR-193b-3p |  |
| hsa-miR-193b* | hsa-miR-193b-5p |  |
| hsa-miR-194 | hsa-miR-194-5p |  |
| hsa-miR-195 | hsa-miR-195-5p |  |
| hsa-miR-196a | hsa-miR-196a-5p |  |
| hsa-miR-196a* | hsa-miR-196a-3p |  |
| hsa-miR-196b | hsa-miR-196b-5p |  |
| hsa-miR-197 | hsa-miR-197-3p |  |
| hsa-miR-198 | hsa-miR-198 |  |
| hsa-miR-199a-5p | hsa-miR-199a-5p |  |
| hsa-miR-199b-5p | hsa-miR-199b-5p |  |
| hsa-miR-19a | hsa-miR-19a-3p |  |
| hsa-miR-19b | hsa-miR-19b-3p |  |
| hsa-miR-19b-1* | hsa-miR-19b-1-5p |  |
| hsa-miR-200a | hsa-miR-200a-3p |  |
| hsa-miR-200a* | hsa-miR-200a-5p |  |
| hsa-miR-200b | hsa-miR-200b-3p |  |
| hsa-miR-200b* | hsa-miR-200b-5p |  |
| hsa-miR-200c | hsa-miR-200c-3p |  |
| hsa-miR-202 | hsa-miR-202-3p |  |
| hsa-miR-203 | hsa-miR-203a-3p |  |
| hsa-miR-204 | hsa-miR-204-5p |  |
| hsa-miR-205 | hsa-miR-205-5p |  |
| hsa-miR-206 | hsa-miR-206 |  |
| hsa-miR-208a | hsa-miR-208a |  |
| hsa-miR-208b | hsa-miR-208b |  |
| hsa-miR-20a | hsa-miR-20a-5p |  |
| hsa-miR-20a* | hsa-miR-20a-3p |  |
| hsa-miR-20b | hsa-miR-20b-5p |  |
| hsa-miR-21 | hsa-miR-21-5p |  |
| hsa-miR-21* | hsa-miR-21-3p |  |
| hsa-miR-210 | hsa-miR-210-3p |  |
| hsa-miR-212 | hsa-miR-212-3p |  |
| hsa-miR-214 | hsa-miR-214-3p |  |
| hsa-miR-215 | hsa-miR-215 |  |
| hsa-miR-217 | hsa-miR-217 |  |
| hsa-miR-218 | hsa-miR-218-5p |  |
| hsa-miR-219-5p | hsa-miR-219-5p |  |
| hsa-miR-22 | hsa-miR-22-3p |  |
| hsa-miR-22* | hsa-miR-22-5p |  |
| hsa-miR-221 | hsa-miR-221-3p |  |
| hsa-miR-221* | hsa-miR-221-5p |  |
| hsa-miR-222 | hsa-miR-222-3p |  |
| hsa-miR-223 | hsa-miR-223-3p |  |
| hsa-miR-223* | hsa-miR-223-5p |  |
| hsa-miR-224 | hsa-miR-224-5p |  |
| hsa-miR-23a | hsa-miR-23a-3p |  |
| hsa-miR-23a* | hsa-miR-23a-5p |  |
| hsa-miR-23b | hsa-miR-23b-3p |  |
| hsa-miR-24 | hsa-miR-24-3p |  |
| hsa-miR-25 | hsa-miR-25-3p |  |
| hsa-miR-26a | hsa-miR-26a-5p |  |
| hsa-miR-26b | hsa-miR-26b-5p |  |
| hsa-miR-26b* | hsa-miR-26b-3p |  |
| hsa-miR-27a | hsa-miR-27a-3p |  |
| hsa-miR-27a* | hsa-miR-27a-5p |  |
| hsa-miR-27b | hsa-miR-27b-3p |  |
| hsa-miR-27b* | hsa-miR-27b-5p |  |
| hsa-miR-28-3p | hsa-miR-28-3p |  |
| hsa-miR-28-5p | hsa-miR-28-5p |  |
| hsa-miR-296-5p | hsa-miR-296-5p |  |
| hsa-miR-298 | hsa-miR-298 |  |
| hsa-miR-299-5p | hsa-miR-299-5p |  |
| hsa-miR-29a | hsa-miR-29a-3p |  |
| hsa-miR-29b | hsa-miR-29b-3p |  |
| hsa-miR-29b-1* | hsa-miR-29b-1-5p |  |
| hsa-miR-29b-2* | hsa-miR-29b-2-5p |  |
| hsa-miR-29c | hsa-miR-29c-3p |  |
| hsa-miR-29c* | hsa-miR-29c-5p |  |
| hsa-miR-301a | hsa-miR-301a-3p |  |
| hsa-miR-301b | hsa-miR-301b |  |
| hsa-miR-302a | hsa-miR-302a-3p |  |
| hsa-miR-302b | hsa-miR-302b-3p |  |
| hsa-miR-302c | hsa-miR-302c-3p |  |
| hsa-miR-30a | hsa-miR-30a-5p |  |
| hsa-miR-30b | hsa-miR-30b-5p |  |
| hsa-miR-30c | hsa-miR-30c-5p |  |
| hsa-miR-30c-2* | hsa-miR-30c-2-3p |  |
| hsa-miR-30d | hsa-miR-30d-5p |  |
| hsa-miR-30d* | hsa-miR-30d-3p |  |
| hsa-miR-30e | hsa-miR-30e-5p |  |
| hsa-miR-30e* | hsa-miR-30e-3p |  |
| hsa-miR-31 | hsa-miR-31-5p |  |
| hsa-miR-32 | hsa-miR-32-5p |  |
| hsa-miR-323-3p | hsa-miR-323a-3p |  |
| hsa-miR-324-3p | hsa-miR-324-3p |  |
| hsa-miR-324-5p | hsa-miR-324-5p |  |
| hsa-miR-326 | hsa-miR-326 |  |
| hsa-miR-328 | hsa-miR-328 |  |
| hsa-miR-330-3p | hsa-miR-330-3p |  |
| hsa-miR-330-5p | hsa-miR-330-5p |  |
| hsa-miR-331-3p | hsa-miR-331-3p |  |
| hsa-miR-335 | hsa-miR-335-5p |  |
| hsa-miR-335* | hsa-miR-335-3p |  |
| hsa-miR-337-5p | hsa-miR-337-5p |  |
| hsa-miR-338-3p | hsa-miR-338-3p |  |
| hsa-miR-339-3p | hsa-miR-339-3p |  |
| hsa-miR-339-5p | hsa-miR-339-5p |  |
| hsa-miR-33a | hsa-miR-33a-5p |  |
| hsa-miR-33b | hsa-miR-33b-5p |  |
| hsa-miR-340 | hsa-miR-340-5p |  |
| hsa-miR-340* | hsa-miR-340-3p |  |
| hsa-miR-342-3p | hsa-miR-342-3p |  |
| hsa-miR-342-5p | hsa-miR-342-5p |  |
| hsa-miR-345 | hsa-miR-345-5p |  |
| hsa-miR-346 | hsa-miR-346 |  |
| hsa-miR-34a | hsa-miR-34a-5p |  |
| hsa-miR-34a* | hsa-miR-34a-3p |  |
| hsa-miR-34b | hsa-miR-34b-3p |  |
| hsa-miR-34b* | hsa-miR-34b-5p |  |
| hsa-miR-34c-5p | hsa-miR-34c-5p |  |
| hsa-miR-361-3p | hsa-miR-361-3p |  |
| hsa-miR-361-5p | hsa-miR-361-5p |  |
| hsa-miR-362-3p | hsa-miR-362-3p |  |
| hsa-miR-362-5p | hsa-miR-362-5p |  |
| hsa-miR-363 | hsa-miR-363-3p |  |
| hsa-miR-365 | hsa-miR-365a-3p |  |
| hsa-miR-367* | hsa-miR-367-5p |  |
| hsa-miR-369-3p | hsa-miR-369-3p |  |
| hsa-miR-370 | hsa-miR-370 |  |
| hsa-miR-371-5p | hsa-miR-371a-5p |  |
| hsa-miR-372 | hsa-miR-372 |  |
| hsa-miR-373 | hsa-miR-373-3p |  |
| hsa-miR-373* | hsa-miR-373-5p |  |
| hsa-miR-374a | hsa-miR-374a-5p |  |
| hsa-miR-374b | hsa-miR-374b-5p |  |
| hsa-miR-375 | hsa-miR-375 |  |
| hsa-miR-376a | hsa-miR-376a-3p |  |
| hsa-miR-376b | hsa-miR-376b |  |
| hsa-miR-376c | hsa-miR-376c |  |
| hsa-miR-378 | hsa-miR-378a-3p |  |
| hsa-miR-378* | hsa-miR-378a-5p |  |
| hsa-miR-379 | hsa-miR-379-5p |  |
| hsa-miR-379* | hsa-miR-379-3p |  |
| hsa-miR-380* | hsa-miR-380-5p |  |
| hsa-miR-381 | hsa-miR-381 |  |
| hsa-miR-382 | hsa-miR-382-5p |  |
| hsa-miR-383 | hsa-miR-383-5p |  |
| hsa-miR-409-3p | hsa-miR-409-3p |  |
| hsa-miR-410 | hsa-miR-410-3p |  |
| hsa-miR-411 | hsa-miR-411-5p |  |
| hsa-miR-412 | hsa-miR-412 |  |
| hsa-miR-421 | hsa-miR-421 |  |
| hsa-miR-422a | hsa-miR-422a |  |
| hsa-miR-423-3p | hsa-miR-423-3p |  |
| hsa-miR-423-5p | hsa-miR-423-5p |  |
| hsa-miR-424 | hsa-miR-424-5p |  |
| hsa-miR-424* | hsa-miR-424-3p |  |
| hsa-miR-425 | hsa-miR-425-5p |  |
| hsa-miR-425* | hsa-miR-425-3p |  |
| hsa-miR-429 | hsa-miR-429 |  |
| hsa-miR-431 | hsa-miR-431-5p |  |
| hsa-miR-431* | hsa-miR-431-3p |  |
| hsa-miR-432 | hsa-miR-432-5p |  |
| hsa-miR-433 | hsa-miR-433 |  |
| hsa-miR-449a | hsa-miR-449a |  |
| hsa-miR-450a | hsa-miR-450a-5p |  |
| hsa-miR-450b-5p | hsa-miR-450b-5p |  |
| hsa-miR-451 | hsa-miR-451a |  |
| hsa-miR-452 | hsa-miR-452-5p |  |
| hsa-miR-453 | hsa-miR-323b-5p |  |
| hsa-miR-454 | hsa-miR-454-3p |  |
| hsa-miR-455-3p | hsa-miR-455-3p |  |
| hsa-miR-483-3p | hsa-miR-483-3p |  |
| hsa-miR-483-5p | hsa-miR-483-5p |  |
| hsa-miR-484 | hsa-miR-484 |  |
| hsa-miR-485-3p | hsa-miR-485-3p |  |
| hsa-miR-485-5p | hsa-miR-485-5p |  |
| hsa-miR-486-3p | hsa-miR-486-3p |  |
| hsa-miR-486-5p | hsa-miR-486-5p |  |
| hsa-miR-487b | hsa-miR-487b |  |
| hsa-miR-488 | hsa-miR-488-3p |  |
| hsa-miR-489 | hsa-miR-489-3p |  |
| hsa-miR-490-3p | hsa-miR-490-3p |  |
| hsa-miR-490-5p | hsa-miR-490-5p |  |
| hsa-miR-491-3p | hsa-miR-491-3p |  |
| hsa-miR-491-5p | hsa-miR-491-5p |  |
| hsa-miR-492 | hsa-miR-492 |  |
| hsa-miR-493 | hsa-miR-493-3p |  |
| hsa-miR-494 | hsa-miR-494 |  |
| hsa-miR-495 | hsa-miR-495 |  |
| hsa-miR-497 | hsa-miR-497-5p |  |
| hsa-miR-498 | hsa-miR-498 |  |
| hsa-miR-499-3p | hsa-miR-499a-3p |  |
| hsa-miR-499-5p | hsa-miR-499a-5p |  |
| hsa-miR-500 | hsa-miR-500a-5p |  |
| hsa-miR-500* | hsa-miR-500a-3p |  |
| hsa-miR-501-3p | hsa-miR-501-3p |  |
| hsa-miR-501-5p | hsa-miR-501-5p |  |
| hsa-miR-502-3p | hsa-miR-502-3p |  |
| hsa-miR-502-5p | hsa-miR-502-5p |  |
| hsa-miR-503 | hsa-miR-503 |  |
| hsa-miR-505 | hsa-miR-505-3p |  |
| hsa-miR-505* | hsa-miR-505-5p |  |
| hsa-miR-508-3p | hsa-miR-508-3p |  |
| hsa-miR-508-5p | hsa-miR-508-5p |  |
| hsa-miR-510 | hsa-miR-510 |  |
| hsa-miR-511 | hsa-miR-511 |  |
| hsa-miR-512-3p | hsa-miR-512-3p |  |
| hsa-miR-513a-3p | hsa-miR-513a-3p |  |
| hsa-miR-513a-5p | hsa-miR-513a-5p |  |
| hsa-miR-513b | hsa-miR-513b |  |
| hsa-miR-513c | hsa-miR-513c-5p |  |
| hsa-miR-515-5p | hsa-miR-515-5p |  |
| hsa-miR-516a-3p | hsa-miR-516a-3p |  |
| hsa-miR-516b | hsa-miR-516b-5p |  |
| hsa-miR-517* | hsa-miR-517-5p |  |
| hsa-miR-517a | hsa-miR-517a-3p |  |
| hsa-miR-517b | hsa-miR-517b-3p |  |
| hsa-miR-517c | hsa-miR-517c-3p |  |
| hsa-miR-518a-3p | hsa-miR-518a-3p |  |
| hsa-miR-518b | hsa-miR-518b |  |
| hsa-miR-518c* | hsa-miR-518c-5p |  |
| hsa-miR-518d-3p | hsa-miR-518d-3p |  |
| hsa-miR-518e | hsa-miR-518e-3p |  |
| hsa-miR-518e* | hsa-miR-518e-5p |  |
| hsa-miR-518f | hsa-miR-518f-3p |  |
| hsa-miR-518f* | hsa-miR-518f-5p |  |
| hsa-miR-519d | hsa-miR-519d |  |
| hsa-miR-519e* | hsa-miR-519e-5p |  |
| hsa-miR-520b | hsa-miR-520b |  |
| hsa-miR-520c-3p | hsa-miR-520c-3p |  |
| hsa-miR-520e | hsa-miR-520e |  |
| hsa-miR-520g | hsa-miR-520g |  |
| hsa-miR-520h | hsa-miR-520h |  |
| hsa-miR-521 | hsa-miR-521 |  |
| hsa-miR-522 | hsa-miR-522-3p |  |
| hsa-miR-523 | hsa-miR-523-3p |  |
| hsa-miR-525-3p | hsa-miR-525-3p |  |
| hsa-miR-532-3p | hsa-miR-532-3p |  |
| hsa-miR-532-5p | hsa-miR-532-5p |  |
| hsa-miR-542-3p | hsa-miR-542-3p |  |
| hsa-miR-542-5p | hsa-miR-542-5p |  |
| hsa-miR-543 | hsa-miR-543 |  |
| hsa-miR-545 | hsa-miR-545-3p |  |
| hsa-miR-548a-3p | hsa-miR-548a-3p |  |
| hsa-miR-548a-5p | hsa-miR-548a-5p |  |
| hsa-miR-548b-5p | hsa-miR-548b-5p |  |
| hsa-miR-548c-5p | hsa-miR-548c-5p |  |
| hsa-miR-548d-5p | hsa-miR-548d-5p |  |
| hsa-miR-550 | hsa-miR-550a-5p |  |
| hsa-miR-550* | hsa-miR-550a-3p |  |
| hsa-miR-551b | hsa-miR-551b-3p |  |
| hsa-miR-556-5p | hsa-miR-556-5p |  |
| hsa-miR-557 | hsa-miR-557 |  |
| hsa-miR-558 | hsa-miR-558 |  |
| hsa-miR-559 | hsa-miR-559 |  |
| hsa-miR-564 | hsa-miR-564 |  |
| hsa-miR-570 | hsa-miR-570-3p |  |
| hsa-miR-571 | hsa-miR-571 |  |
| hsa-miR-572 | hsa-miR-572 |  |
| hsa-miR-574-3p | hsa-miR-574-3p |  |
| hsa-miR-574-5p | hsa-miR-574-5p |  |
| hsa-miR-575 | hsa-miR-575 |  |
| hsa-miR-576-3p | hsa-miR-576-3p |  |
| hsa-miR-576-5p | hsa-miR-576-5p |  |
| hsa-miR-577 | hsa-miR-577 |  |
| hsa-miR-578 | hsa-miR-578 |  |
| hsa-miR-579 | hsa-miR-579-3p |  |
| hsa-miR-580 | hsa-miR-580 |  |
| hsa-miR-582-3p | hsa-miR-582-3p |  |
| hsa-miR-582-5p | hsa-miR-582-5p |  |
| hsa-miR-583 | hsa-miR-583 |  |
| hsa-miR-584 | hsa-miR-584-5p |  |
| hsa-miR-585 | hsa-miR-585 |  |
| hsa-miR-590-5p | hsa-miR-590-5p |  |
| hsa-miR-591 | hsa-miR-591 |  |
| hsa-miR-593 | hsa-miR-593-3p |  |
| hsa-miR-593* | hsa-miR-593-5p |  |
| hsa-miR-595 | hsa-miR-595 |  |
| hsa-miR-596 | hsa-miR-596 |  |
| hsa-miR-598 | hsa-miR-598 |  |
| hsa-miR-601 | hsa-miR-601 |  |
| hsa-miR-603 | hsa-miR-603 |  |
| hsa-miR-605 | hsa-miR-605 |  |
| hsa-miR-608 | hsa-miR-608 |  |
| hsa-miR-609 | hsa-miR-609 |  |
| hsa-miR-610 | hsa-miR-610 |  |
| hsa-miR-612 | hsa-miR-612 |  |
| hsa-miR-614 | hsa-miR-614 |  |
| hsa-miR-615-3p | hsa-miR-615-3p |  |
| hsa-miR-616* | hsa-miR-616-5p |  |
| hsa-miR-617 | hsa-miR-617 |  |
| hsa-miR-618 | hsa-miR-618 |  |
| hsa-miR-619 | hsa-miR-619 |  |
| hsa-miR-621 | hsa-miR-621 |  |
| hsa-miR-622 | hsa-miR-622 |  |
| hsa-miR-624* | hsa-miR-624-5p |  |
| hsa-miR-625 | hsa-miR-625-5p |  |
| hsa-miR-625* | hsa-miR-625-3p |  |
| hsa-miR-627 | hsa-miR-627-5p |  |
| hsa-miR-628-3p | hsa-miR-628-3p |  |
| hsa-miR-628-5p | hsa-miR-628-5p |  |
| hsa-miR-629 | hsa-miR-629-5p |  |
| hsa-miR-629* | hsa-miR-629-3p |  |
| hsa-miR-630 | hsa-miR-630 |  |
| hsa-miR-631 | hsa-miR-631 |  |
| hsa-miR-632 | hsa-miR-632 |  |
| hsa-miR-634 | hsa-miR-634 |  |
| hsa-miR-635 | hsa-miR-635 |  |
| hsa-miR-636 | hsa-miR-636 |  |
| hsa-miR-637 | hsa-miR-637 |  |
| hsa-miR-638 | hsa-miR-638 |  |
| hsa-miR-639 | hsa-miR-639 |  |
| hsa-miR-640 | hsa-miR-640 |  |
| hsa-miR-642 | hsa-miR-642a-5p |  |
| hsa-miR-643 | hsa-miR-643 |  |
| hsa-miR-644 | hsa-miR-644a |  |
| hsa-miR-645 | hsa-miR-645 |  |
| hsa-miR-646 | hsa-miR-646 |  |
| hsa-miR-647 | hsa-miR-647 |  |
| hsa-miR-648 | hsa-miR-648 |  |
| hsa-miR-649 | hsa-miR-649 |  |
| hsa-miR-652 | hsa-miR-652-3p |  |
| hsa-miR-654-3p | hsa-miR-654-3p |  |
| hsa-miR-654-5p | hsa-miR-654-5p |  |
| hsa-miR-657 | hsa-miR-657 |  |
| hsa-miR-658 | hsa-miR-658 |  |
| hsa-miR-659 | hsa-miR-659-3p |  |
| hsa-miR-660 | hsa-miR-660-5p |  |
| hsa-miR-662 | hsa-miR-662 |  |
| hsa-miR-663 | hsa-miR-663a |  |
| hsa-miR-665 | hsa-miR-665 |  |
| hsa-miR-668 | hsa-miR-668 |  |
| hsa-miR-671-3p | hsa-miR-671-3p |  |
| hsa-miR-671-5p | hsa-miR-671-5p |  |
| hsa-miR-675 | hsa-miR-675-5p |  |
| hsa-miR-7 | hsa-miR-7-5p |  |
| hsa-miR-7-1* | hsa-miR-7-1-3p |  |
| hsa-miR-744 | hsa-miR-744-5p |  |
| hsa-miR-744* | hsa-miR-744-3p |  |
| hsa-miR-760 | hsa-miR-760 |  |
| hsa-miR-765 | hsa-miR-765 |  |
| hsa-miR-766 | hsa-miR-766-3p |  |
| hsa-miR-769-3p | hsa-miR-769-3p |  |
| hsa-miR-769-5p | hsa-miR-769-5p |  |
| hsa-miR-770-5p | hsa-miR-770-5p |  |
| hsa-miR-874 | hsa-miR-874 |  |
| hsa-miR-875-5p | hsa-miR-875-5p |  |
| hsa-miR-877 | hsa-miR-877-5p |  |
| hsa-miR-885-5p | hsa-miR-885-5p |  |
| hsa-miR-886-3p | hsa-miR-886-3p |  |
| hsa-miR-886-5p | hsa-miR-886-5p |  |
| hsa-miR-887 | hsa-miR-887 |  |
| hsa-miR-888* | hsa-miR-888-3p |  |
| hsa-miR-891a | hsa-miR-891a-5p |  |
| hsa-miR-892b | hsa-miR-892b |  |
| hsa-miR-9 | hsa-miR-9-5p |  |
| hsa-miR-9* | hsa-miR-9-3p |  |
| hsa-miR-921 | hsa-miR-921 |  |
| hsa-miR-924 | hsa-miR-924 |  |
| hsa-miR-92a | hsa-miR-92a-3p |  |
| hsa-miR-92a-1* | hsa-miR-92a-1-5p |  |
| hsa-miR-92a-2* | hsa-miR-92a-2-5p |  |
| hsa-miR-92b | hsa-miR-92b-3p |  |
| hsa-miR-93 | hsa-miR-93-5p |  |
| hsa-miR-93* | hsa-miR-93-3p |  |
| hsa-miR-934 | hsa-miR-934 |  |
| hsa-miR-936 | hsa-miR-936 |  |
| hsa-miR-939 | hsa-miR-939-5p |  |
| hsa-miR-940 | hsa-miR-940 |  |
| hsa-miR-942 | hsa-miR-942 |  |
| hsa-miR-943 | hsa-miR-943 |  |
| hsa-miR-95 | hsa-miR-95-3p |  |
| hsa-miR-96 | hsa-miR-96-5p |  |
| hsa-miR-96* | hsa-miR-96-3p |  |
| hsa-miR-98 | hsa-miR-98 |  |
| hsa-miR-99a | hsa-miR-99a-5p |  |
| hsa-miR-99a* | hsa-miR-99a-3p |  |
| hsa-miR-99b | hsa-miR-99b-5p |  |
| hsa-miR-99b* | hsa-miR-99b-3p |  |

| **49 miRNAs included in array and used for in silico validation** |
| --- |
| hsa-let-7e-5p |
| hsa-miR-124-3p |
| hsa-miR-125b-1-3p |
| hsa-miR-125b-2-3p |
| hsa-miR-126-5p |
| hsa-miR-132-3p |
| hsa-miR-135b-5p |
| hsa-miR-138-5p |
| hsa-miR-141-3p |
| hsa-miR-142-3p |
| hsa-miR-142-5p |
| hsa-miR-144-3p |
| hsa-miR-146a-5p |
| hsa-miR-146b-5p |
| hsa-miR-150-5p |
| hsa-miR-151a-3p |
| hsa-miR-152-3p |
| hsa-miR-16-2-3p |
| hsa-miR-181a-2-3p |
| hsa-miR-18a-5p |
| hsa-miR-195-5p |
| hsa-miR-196a-5p |
| hsa-miR-199b-5p |
| hsa-miR-20a-5p |
| hsa-miR-218-5p |
| hsa-miR-221-3p |
| hsa-miR-221-5p |
| hsa-miR-222-3p |
| hsa-miR-223-3p |
| hsa-miR-30a-5p |
| hsa-miR-30c-2-3p |
| hsa-miR-326 |
| hsa-miR-335-5p |
| hsa-miR-337-5p |
| hsa-miR-339-5p |
| hsa-miR-34a-5p |
| hsa-miR-378a-3p |
| hsa-miR-431-5p |
| hsa-miR-449a |
| hsa-miR-483-3p |
| hsa-miR-490-5p |
| hsa-miR-500a-3p |
| hsa-miR-501-3p |
| hsa-miR-532-5p |
| hsa-miR-628-5p |
| hsa-miR-654-3p |
| hsa-miR-769-3p |
| hsa-miR-95-3p |
| hsa-miR-99b-5p |
